# Author Correction: Alkali cation-induced cathodic corrosion in Cu electrocatalysts

**DOI:** 10.1038/s41467-024-50241-z

**Published:** 2024-07-19

**Authors:** Shikai Liu, Yuheng Li, Di Wang, Shibo Xi, Haoming Xu, Yulin Wang, Xinzhe Li, Wenjie Zang, Weidong Liu, Mengyao Su, Katherine Yan, Adam C. Nielander, Andrew B. Wong, Jiong Lu, Thomas F. Jaramillo, Lei Wang, Pieremanuele Canepa, Qian He

**Affiliations:** 1 Department of Material Science and Engineering, College of Design and Engineering, National University of Singapore, 9 Engineering Drive 1, EA #03-09, Singapore, 117575 Singapore; 2 Department of Chemical and Biomolecular Engineering, College of Design and Engineering, National University of Singapore, 4 Engineering Drive 4, E5 #02-29, Singapore, 117585 Singapore; 3 Institute of Sustainability for Chemicals, Energy and Environment (ISCE2), Agency for Science, Technology and Research (A*STAR), 1 Pesek Road Jurong Island, Singapore, 627833 Singapore; 4 Department of Chemistry, National University of Singapore, 12 Science Drive 3, Singapore, 117543 Singapore; 5 SUNCAT Center for Interface Science and Catalysis, Department of Chemical Engineering, Stanford University, Stanford, CA 94305 USA; 6 Centre for Hydrogen Innovations, National University of Singapore, E8, 1 Engineering Drive 3, Singapore, 117580 Singapore; 7 SUNCAT Center for Interface Science and Catalysis, SLAC National Accelerator Laboratory, Menlo Park, CA 94025 USA

**Keywords:** Electrocatalysis, Chemical engineering, Catalytic mechanisms

Correction to: *Nature Communications* 10.1038/s41467-024-49492-7, published online 13 June 2024

In the Acknowledgements section of the article “K.Y., A.C.N., and T.F.J. acknowledge support for evaluating Cu corrosion using mass spectrometry from the Liquid Sunlight Alliance, which is supported by the Basic Energy Sciences program of the US Department of Energy Office of Science through the Fuels from Sunlight Hub under award number DE-SC0021266.” was missing. The original article has been corrected.

