# Supplementary material for: Alkali cation-induced cathodic corrosion in Cu electrocatalysts
Source: Nat Commun. 2024 Jun 13;15:5080. doi: 10.1038/s41467-024-49492-7 (PMC11176167; doi:10.1038/s41467-024-49492-7)
Supplement: Supplementary file 1 — Supplementary Information [file 41467_2024_49492_MOESM1_ESM.pdf]

Supporting Information

Alkali Cation Induced Cathodic Corrosion in Cu Electrocatalysts

Shikai Liu,[1,†] Yuheng Li,[1,†] Di Wang,[2,†] Shibo Xi,[3,*] Haoming Xu,[4] Yulin Wang,[4] Xinzhe Li,[1] Wenjie Zang,[1], Weidong Liu,[1] Mengyao Su,[1] Katherine Yan,[5] Adam C Nielander,[5] Andrew B. Wong,[1,2,6] Jiong Lu,[4,6] Thomas F. Jaramillo,[5,7] Lei Wang,[2,6,*] Pieremanuele Canepa[1,2,*] and Qian He[1,6,*]

1 Department of Material Science and Engineering, College of Design and Engineering, National University of Singapore, 9 Engineering Drive 1, EA #03-09, 117575, Singapore.

2 Department of Chemical and Biomolecular Engineering, College of Design and Engineering, National University of Singapore, 4 Engineering Drive 4, E5 #02-29, 117585, Singapore.

3 Institute of Sustainability for Chemicals, Energy and Environment (ISCE^2), Agency for Science, Technology and Research (A*STAR), 1 Pesek Road Jurong Island, 627833, Singapore.

4 Department of Chemistry, National University of Singapore, 12 Science Drive 3, 117543, Singapore.

5 SUNCAT Center for Interface Science and Catalysis, Department of Chemical Engineering, Stanford University, Stanford, CA 94305, USA.

6 Centre for Hydrogen Innovations, National University of Singapore, E8, 1 Engineering Drive 3, 117580, Singapore.

7 SUNCAT Center for Interface Science and Catalysis, SLAC National Accelerator Laboratory, Menlo Park, CA 94025, USA.

† These authors contributed equally

*corresponding authors: Qian He, mseheq@nus.edu.sg; Pieremanuele Canepa, pcanepa@nus.edu.sg; Lei Wang, wanglei8@nus.edu.sg; Shibo Xi, xi_shibo@isce2.a-star.edu.sg

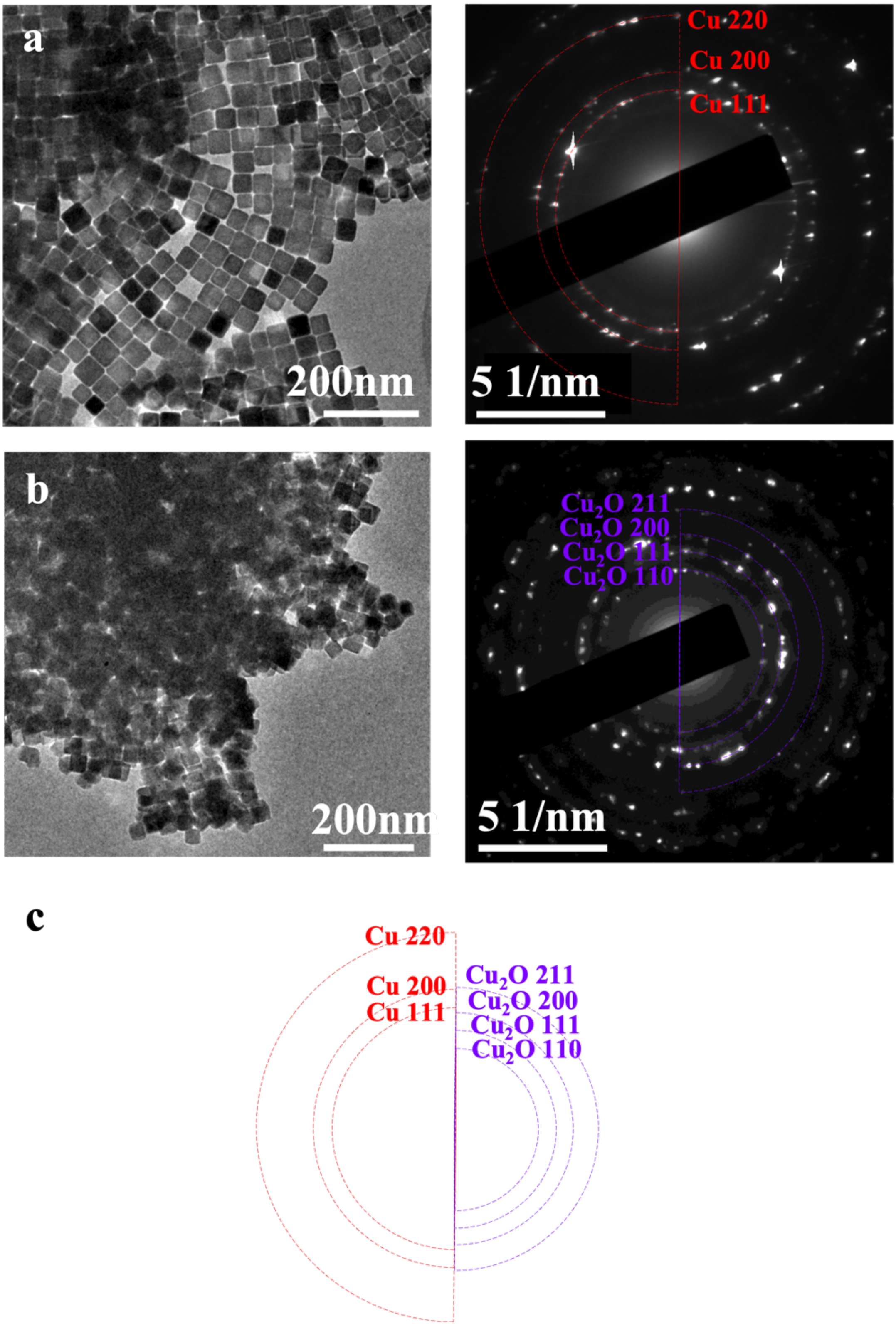

Figure S1 Representative TEM bright field image and the corresponding electron diffraction patterns from **a** Cu nanocubes and **b** Cu_2O nanocubes. **c**. Comparison between the electron diffraction of Cu (red) and Cu_2O nanocubes (purple).

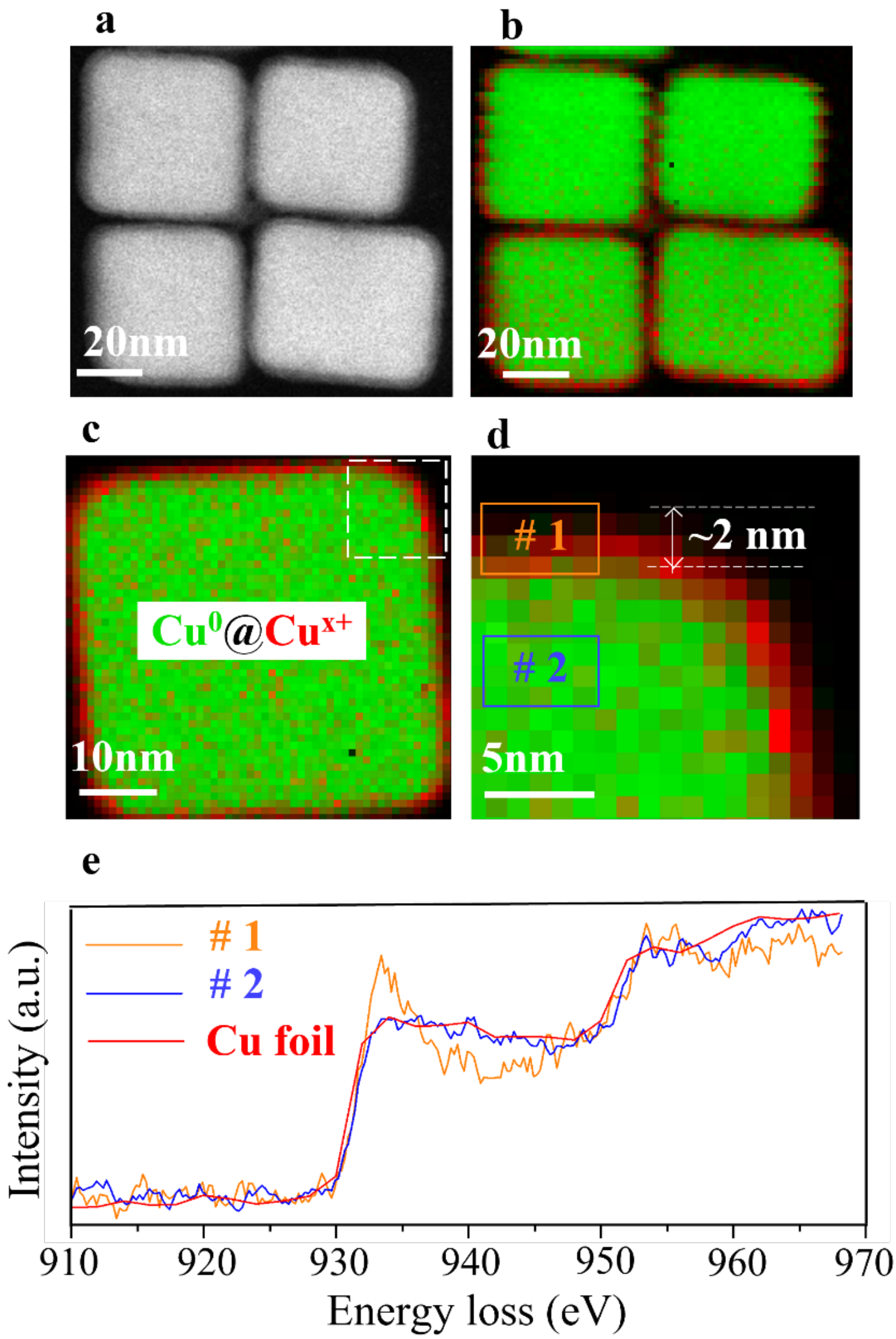

Figure S2 From fresh Cu cubes saved in hexane overnight and exposed to air for less than 10mins. (a,b) HADFF image and STEM-EELS map of metallic (Cu^0) and cationic Cu^{d+} distribution. (c,d) STEM-EELS map and enlarged image from one corner of the cube (e) the EEL spectra from regions #1 and #2 highlighted in (d) and a Cu reference spectrum.

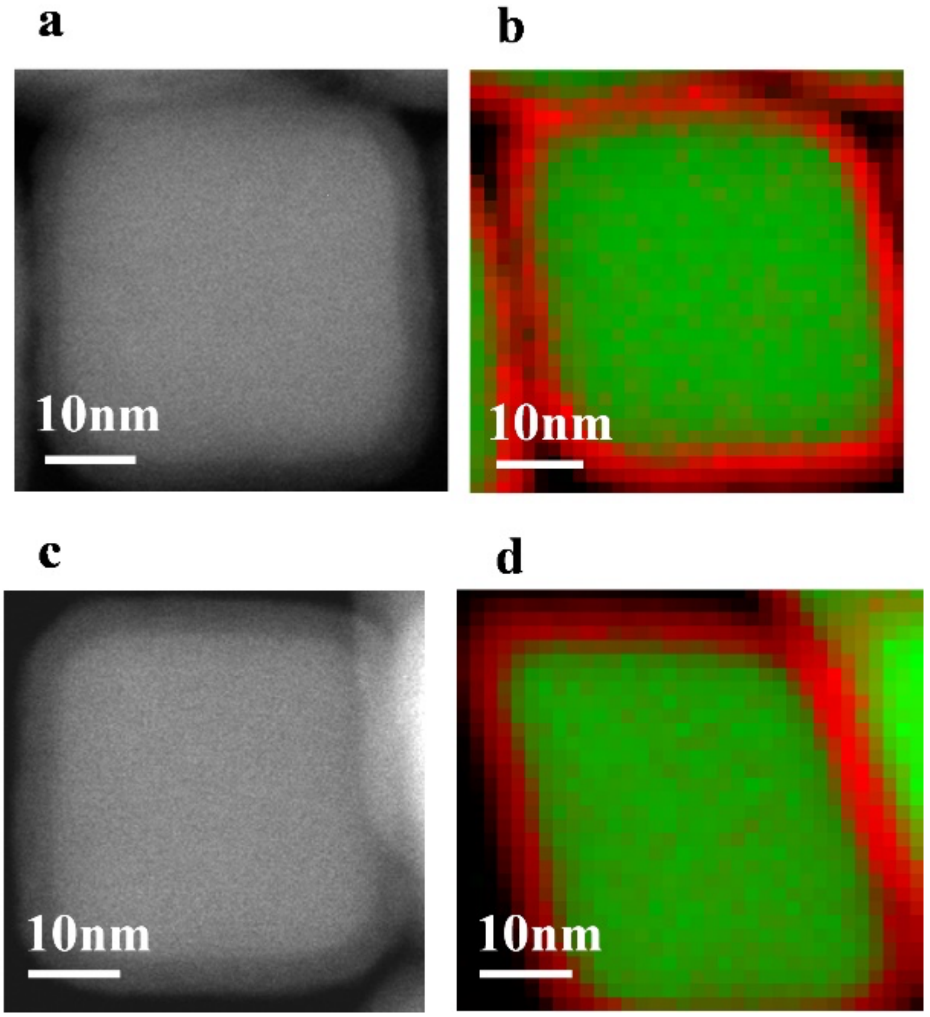

Figure S3 From Cu cubes saved in hexane for 3 days and exposed to air for about 3 hrs. (a-d) HADFF image and STEM-EELS map of metallic (Cu^0) and cationic Cu^{d+} distribution.

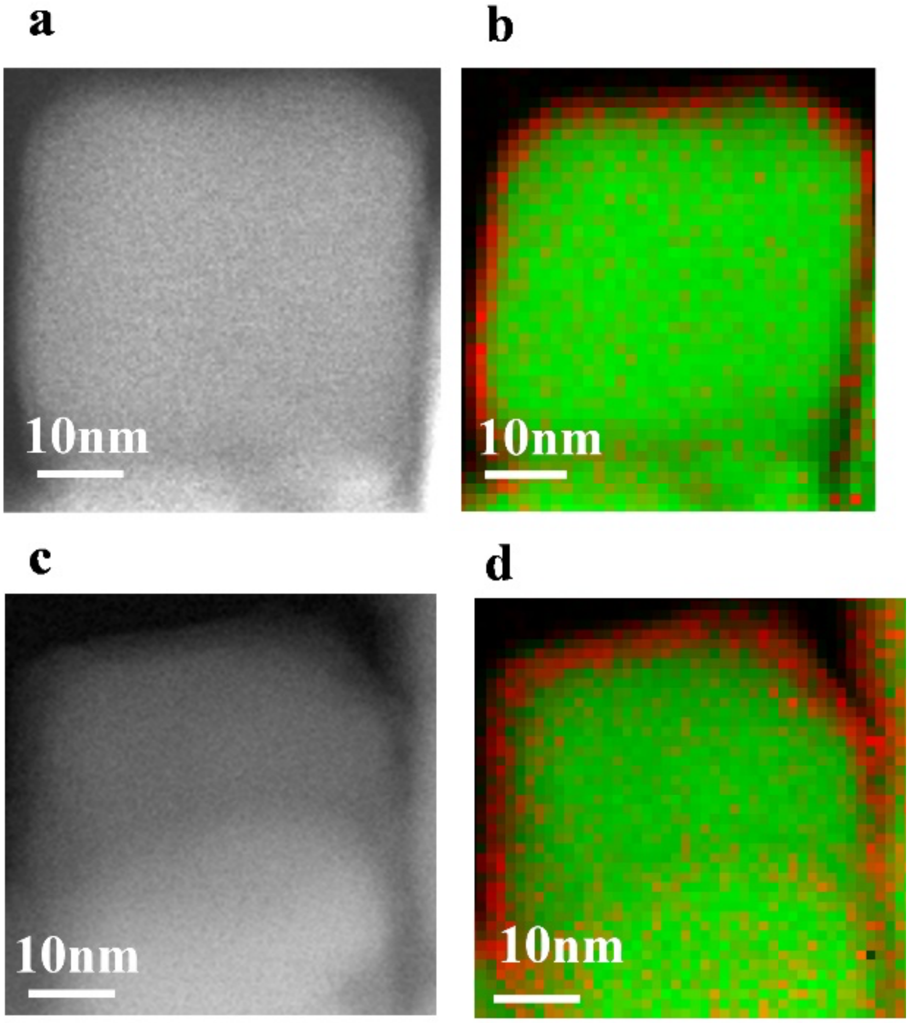

Figure S4 From Cu cubes saved in hexane for 1 days and exposed to air for about 3 days. (a-d) HADFF image and STEM-EELS map of metallic (Cu^0) and cationic Cu^{d+} distribution.

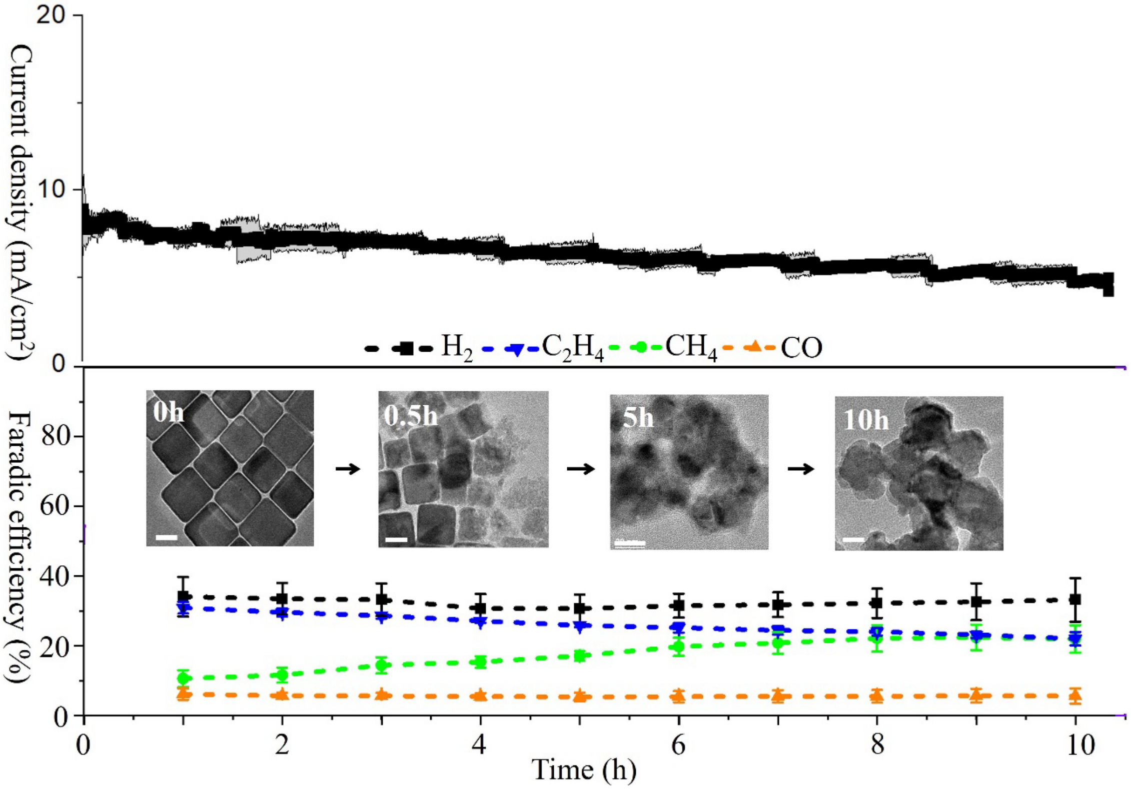

Figure S5 The current density and gas phase products of the Cu nanocubes at -1.1 V_{RHE} over 10h CO_2RR (**Table S1,** entry 1). Shaded areas of the current profile show standard deviations from three independent measurements. The error bars of the faradic efficiency represents standard error of mean from three independent measurements. Inset: Representative TEM bright field images of Cu nanocubes at different stages of the CO_2RR time. Scale bars represent 20nm.

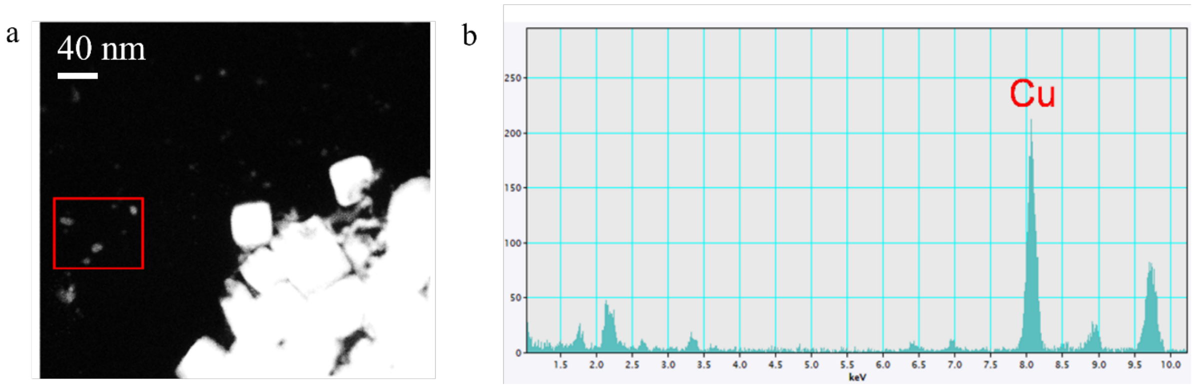

Figure S6 a Representative HAADF-STEM image of Cu cubes after 30min CO_2RR at -1.1V_{RHE} (**Table S1,** entry 2) and **b** the corresponding EDS from small particles (red square region in **a**) that were not there in the fresh samples.

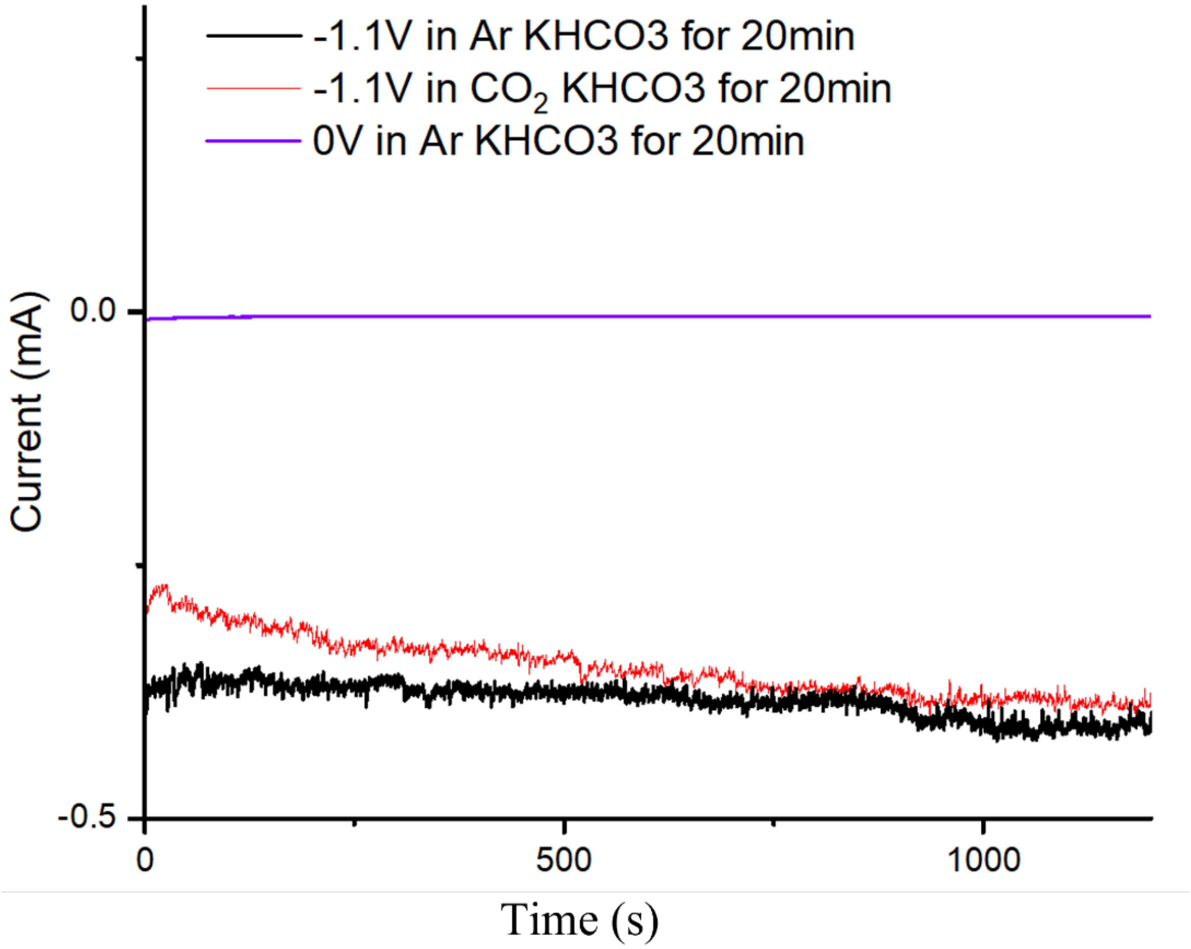

Figure S7 Current density recorded during IL-TEM in CO_2 saturated 0.1M $KHCO_3$ (red, **Table S1,** entry 2), Ar saturated 0.1M KHCO3 **Table S1,** entry 3) at about -1.1V_{RHE} and Ar saturated 0.1M $KHCO_3$ (purple, **Table S1,** entry 4) at 0V_{RHE}.

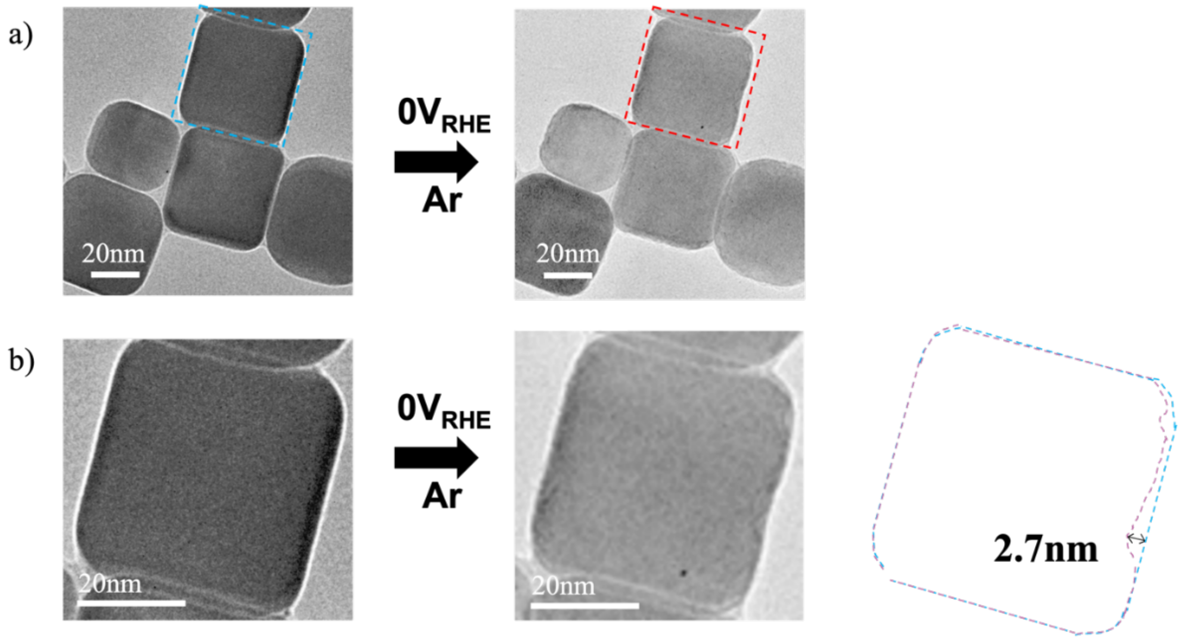

Figure S8 a Morphology evolution of Cu cubes imaged with IL-TEM before and after 20min reaction at 0V$_{RHE}$ in Ar saturated 0.1M $KHCO_3$ (**Table S1,** entry 4). **b** A closed-up look of one particle selected from a and comparison of their outlines before (blue) and after(pink) the reaction, showing that the surface change was about 2.7 nm, which is within the thickness of surface oxide layer. The current recorded during the IL-TEM experiment can be found in **Figures S7.**

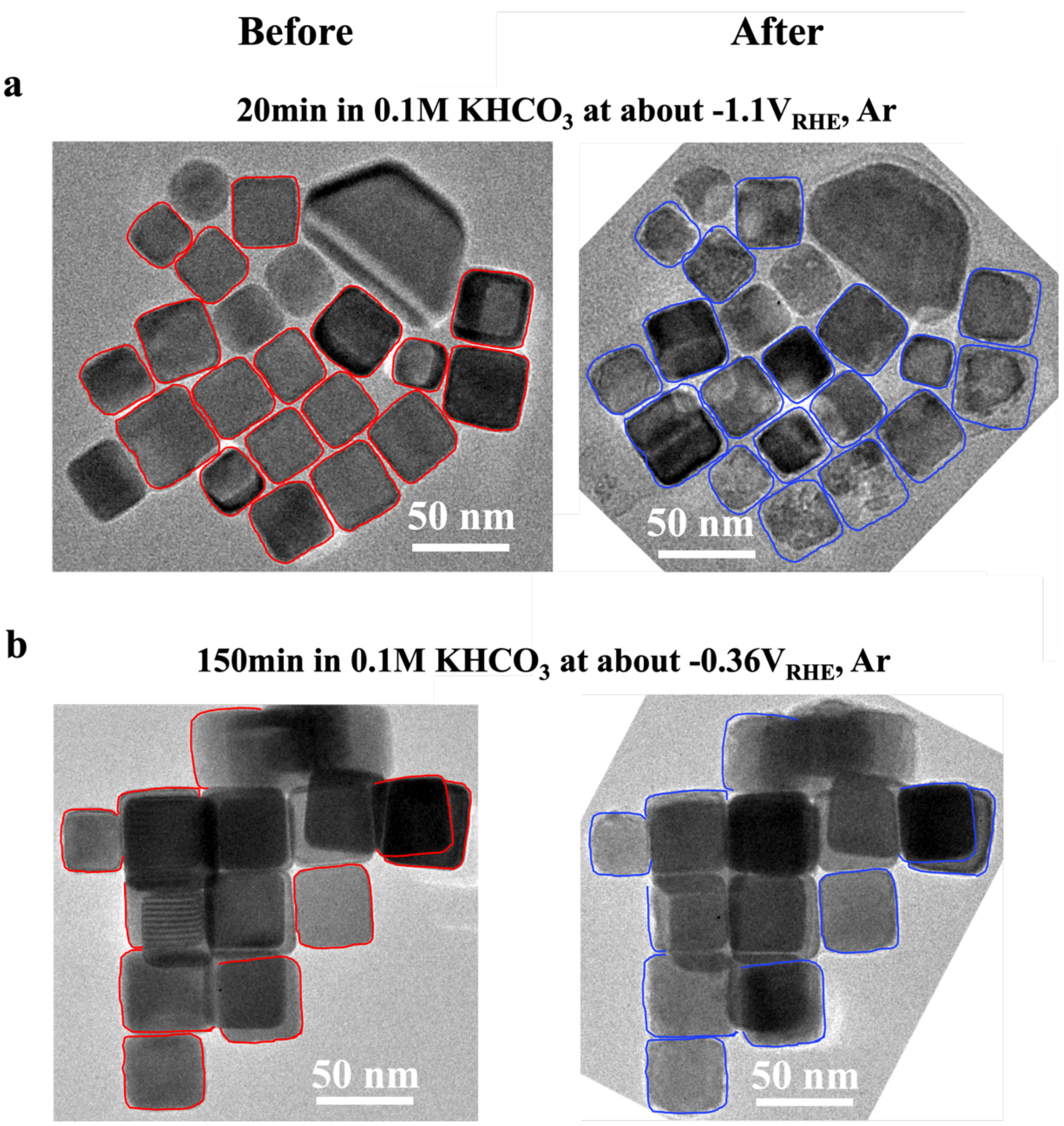

Figure S9 Representative cases showing Cu NCs before and after (**a**) 20min in Ar-saturated 0.1M $KHCO_3$ at about -1.1V (**Table S1,** entry 3) (**b**) 150min in Ar-saturated 0.1M $KHCO_3$ at about -0.36V (**Table S1,** entries 20 and 25) imaged by IL-TEM. The blue profiles represent the outlines acquired from the particles before the reaction. The bold blue lines were drawn to measure the maximum difference between the Cu cubes in the projected images before and after the reaction and represented as the maximum corrosion depth in **Fig S10**. To mitigate the potential influence of particle orientation on the projected shapes, Cu cubes aligned approximately to the [100] zone axis, exhibiting a cubic shape with averaged lengths in the TEM images, were predominantly selected for the measurements.

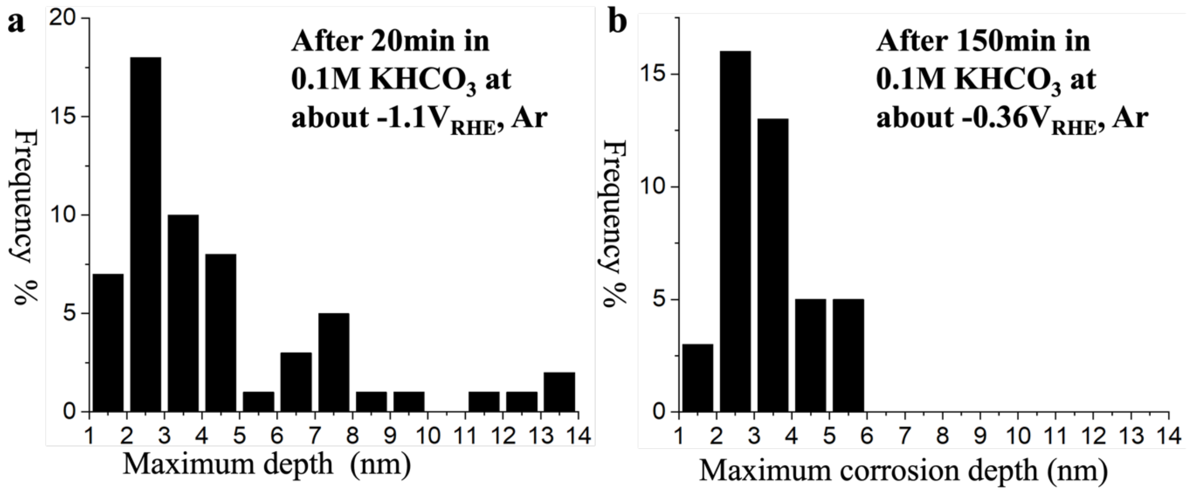

Figure S10 Statistical distribution of maximum depth of morphological changes of the samples shown in ***Figure S9***, manually measured (as in the case of **Figures 1** and **2**) from IL-TEM images of Cu NCs (**a**) after 20min in 0.1M $KHCO_3$ at about -1.1V_{RHE}, Ar; (**b**) after 150min in 0.1M $KHCO_3$ at about -0.36V_{RHE}, Ar. The sample size is about 40 particles in each case.

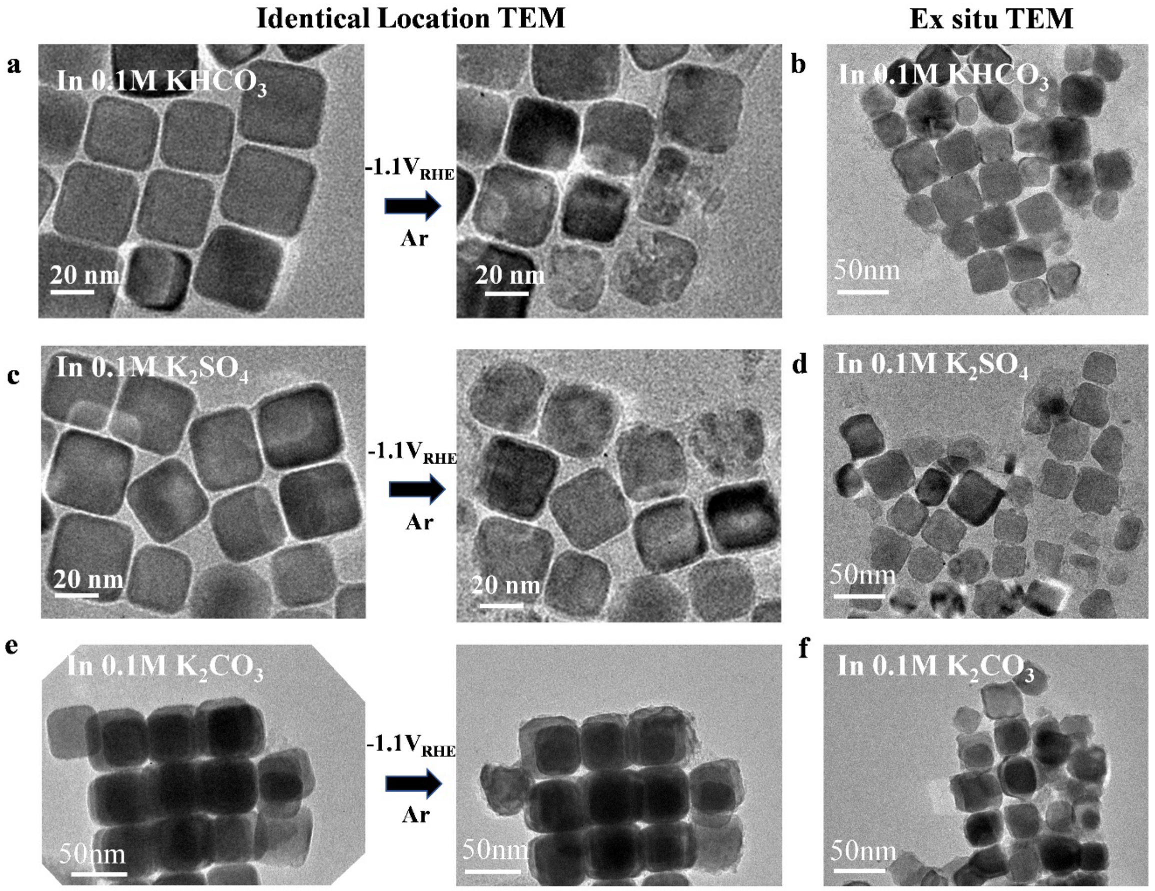

Figure S11 IL-TEM and the corresponding *ex situ* TEM results before and after 20min reaction at -1.1V_{RHE} in Ar saturated **a, b** 0.1M $KHCO_3$ (**Table S1,** entry 3), **c, d** 0.1M K_2SO_4 (**Table S1,** entry 5) and **e,f** 0.1M K_2CO_3 (**Table S1,** entry 6).

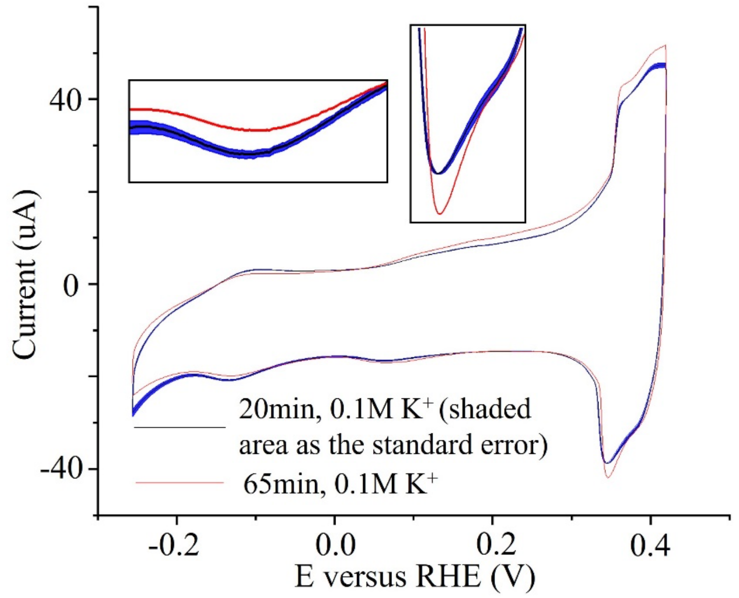

Figure S12 Voltammetric profiles of the Cu nanocubes recorded after 20 mins with standard errors from three independent measurements (blue shaded area) and 65 mins (red curve) of reactions at about $-1.05V_{RHE}$ in 0.1M $KHCO_3$. We conducted three separate CV experiments using three electrodes of Cu nanocubes prepared at the same batch and treated under identical conditions: at -1.1V for 20 minutes in 0.1M $KHCO_3$ to get the voltammetry with error bar. It's evident that the results exhibit high consistency, and the CV of the 65-minute treated sample diverges significantly from the 20min benchmark, therefore agreeing with the observations in Figure 2e.

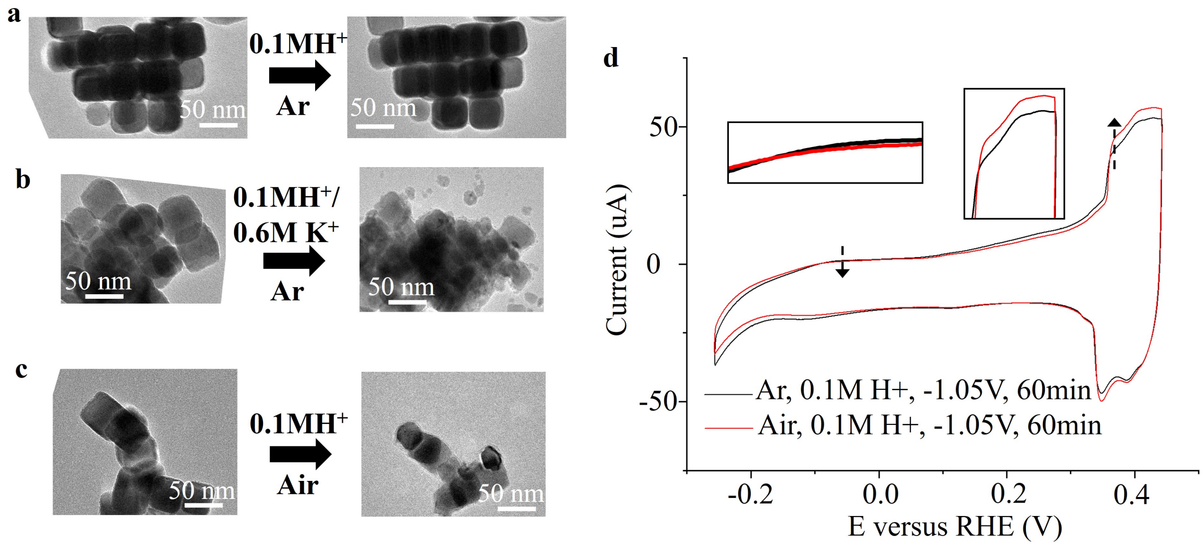

Figure S13 Representative IL-TEM bright field images of Cu nanocubes before and after 30 mins of reaction at about -1.1V_{RHE} in Ar saturated 0.05M H_2SO_4 (**Table S1,** entry 7) (**a**), 0.05M H_2SO_4& 0.3M K_2SO_4 (**Table S1,** entry 8) (**b**) and Air saturated 0.05M H_2SO_4 (**Table S1,** entry 9) (**c**), showing that dissolution of copper was more evident with presence of K^+ and air. (**d**) Voltammetric profiles of the Cu nanocubes recorded after 60 mins reactions at about -1.05V_{RHE} in Ar saturated 0.05M H_2SO_4 (black curve, **Table S1,** entry 7) and Air saturated 0.05M H_2SO_4 (red curve, **Table S1,** entry 9), indicating that presence of oxygen promotes the generation of defects feature under cathodic potential.

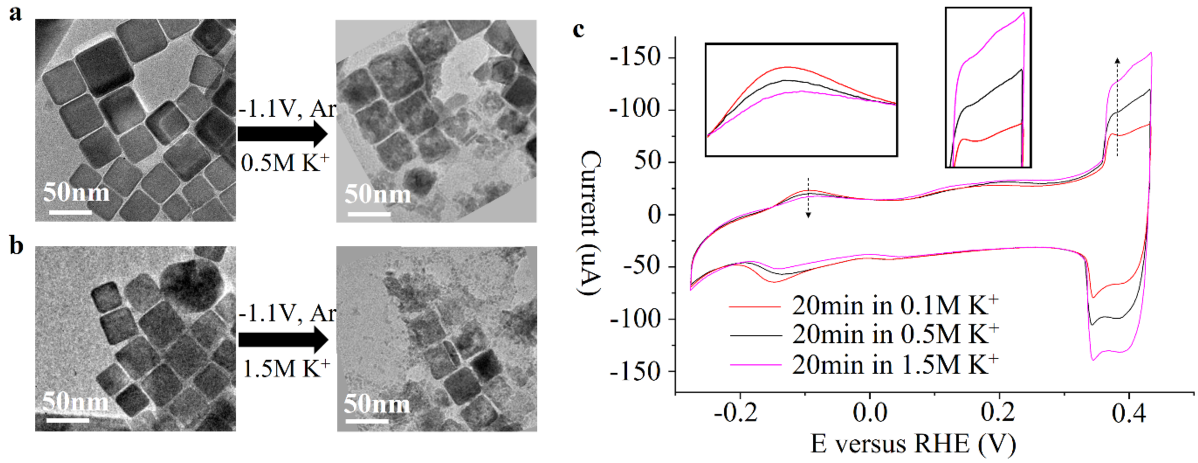

Figure S14 Representative IL-TEM bright field images of Cu nanocubes imaged before and after 20 mins of reaction at about -1.1V_{RHE} in (**a**) 0.5M (**Table S1,** entry 10) and (**b**) 1.5M Ar-saturated $KHCO_3$ (**Table S1,** entry 11). (**c**) Voltammetric profiles of the Cu nanocubes were recorded after 20 mins of reaction at about -1.1 V_{RHE} in Ar-saturated $KHCO_3$ electrolyte with K+ concentration of 0.1 M (red curve, **Table S1,** entry 3)), 0.5 M (grey curve, **Table S1,** entry 10), 1.5 M (pink curve, **Table S1,** entry 11). The arrows highlight that the facet feature was suppressed while the defects feature was enhanced with increasing K^+ concentration in the electrolyte.

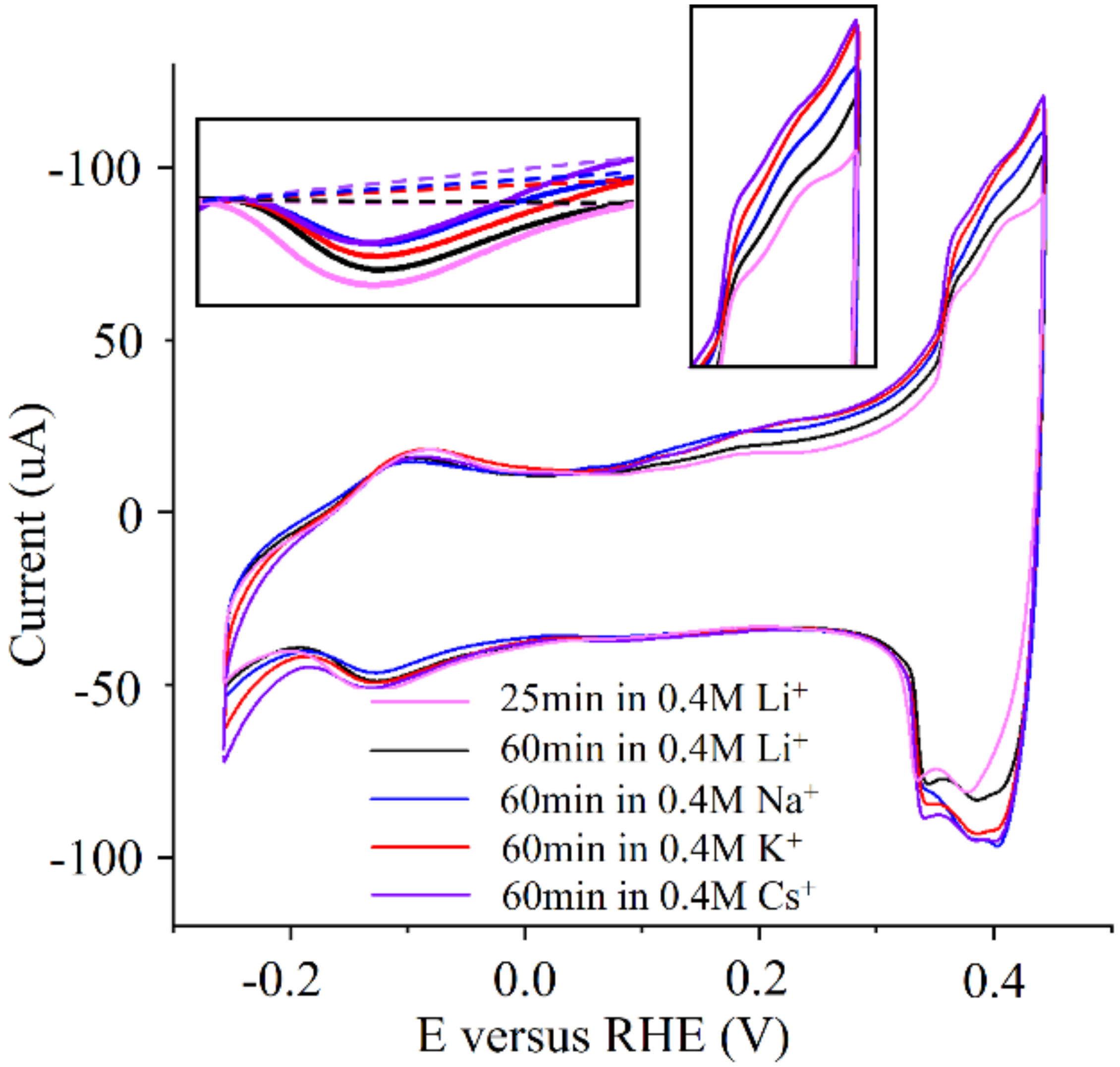

Figure S15 Voltammetric profiles of the Cu cubes recorded after 20min reaction at about -1.1V_{RHE} in 0.2M Li_2SO_4 (red curve) and 60min at around -1.1V_{RHE} in 0.2M Li_2SO_4/0.2M Na_2SO_4/0.2M K_2SO_4/0.2M Cs_2SO_4 (**Table S1,** entry 12-15). Additional morphology change was observed after 60min at -1.1V_{RHE} compared with 25min case in 0.2M Li_2SO_4, indicating the existence of cathodic corrosion with Li+ in electrolyte. The facet feature was suppressed while defects feature was enhanced with larger cations (Na^+/K^+/Cs^+) in electrolyte. The anodic peak corresponding to the {100} facets in the inset was aligned to the same position to give a better visual comparison.

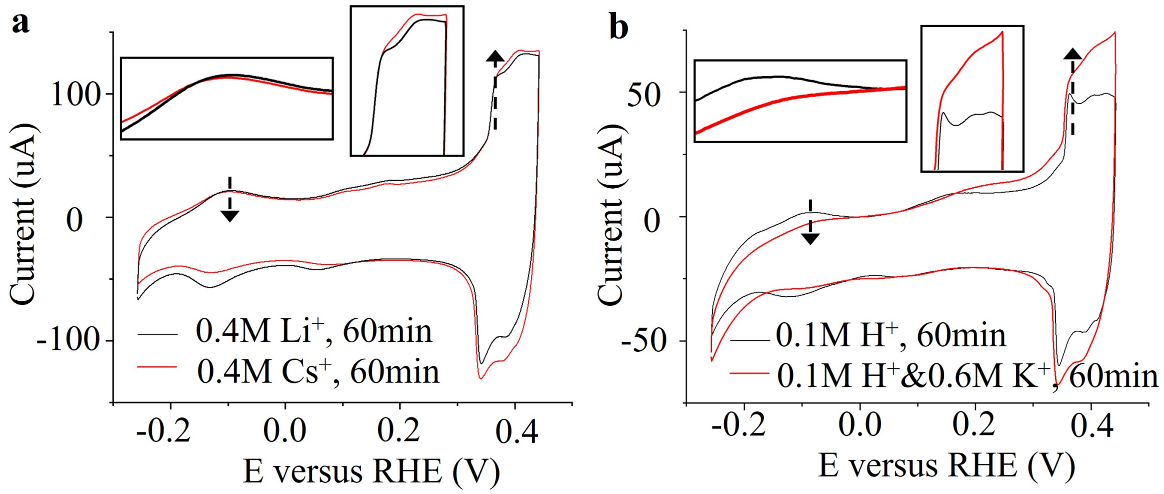

Figure S16 a Voltammetric profiles of the Cu cubes recorded after 60min at constant current of -8.5mA in 0.2M Li_2SO_4 (-1.27V_{RHE}) and Cs_2SO_4 (-1.1V_{RHE}). **b** Voltammetric profiles of the Cu cubes recorded after 60min at constant current of -7.5mA in 0.05M H_2SO_4 (-0.92V_{RHE}) and 0.05M H_2SO_4 & 0.3M K_2SO_4 (-1.5V_{RHE}) showing that the reconstruction of Cu depends on the alkaline cations in electrolyte, rather than the reaction rate (**Table S1,** entries 16-19).

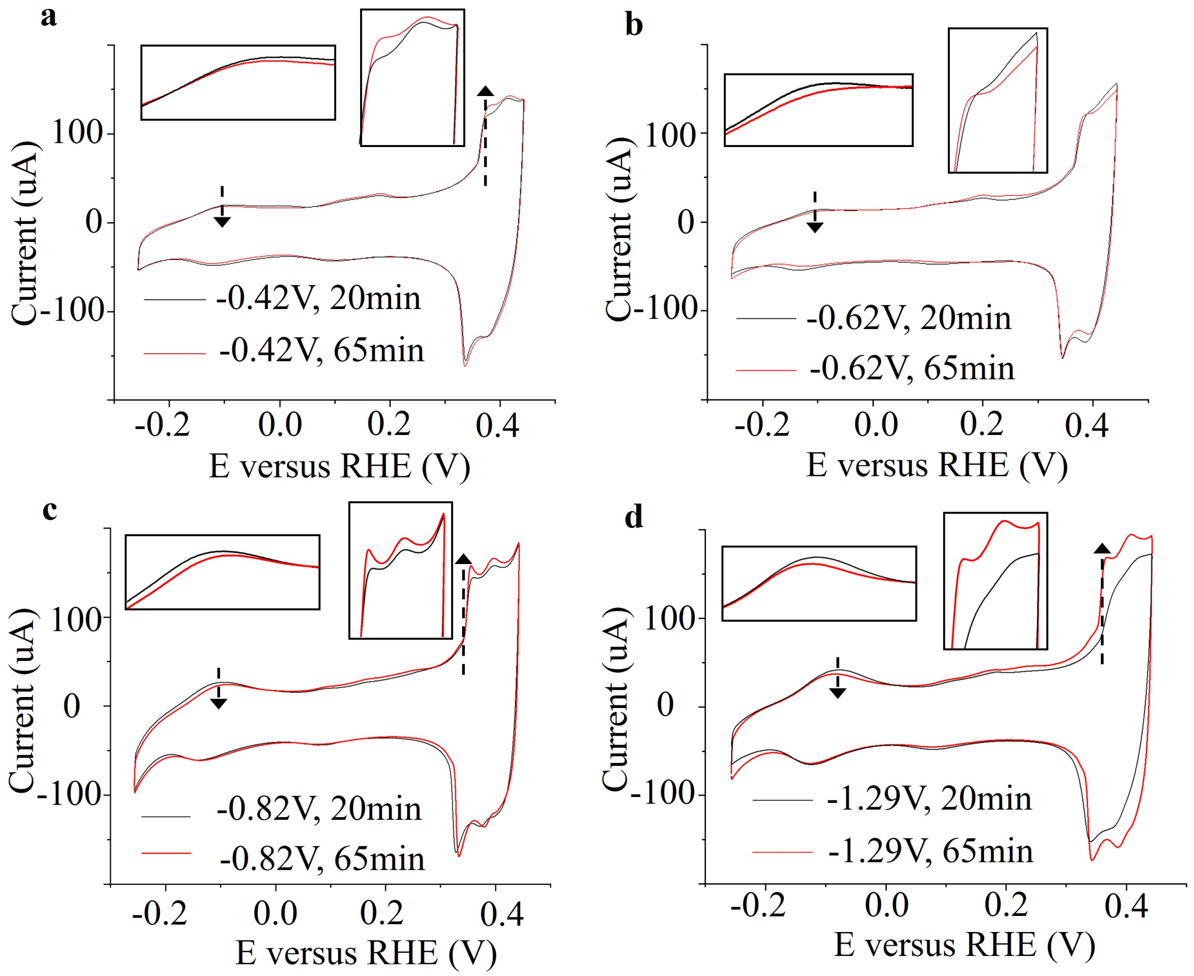

Figure S17 Voltammetric profiles recorded in 0.1M NaOH of the Cu nanocubes after 20min treatment (black curve) and after 65min (red curve) treatment at **a** -0.42 V_{RHE} and **b** -0.62 V_{RHE} **c** -0.82 V_{RHE} **d** -1.29 V_{RHE} in Ar saturated 0.1M $KHCO_3$, showing that the facet feature of copper is more stabilized at -0.42 V_{RHE} than at -0.62 V_{RHE} and -0.82 V_{RHE} and defects related feature has apparently more significant increase at -1.29V_{RHE} than at -0.62 V_{RHE} and -0.82 V_{RHE} **(Table S1,** entry 21-24).

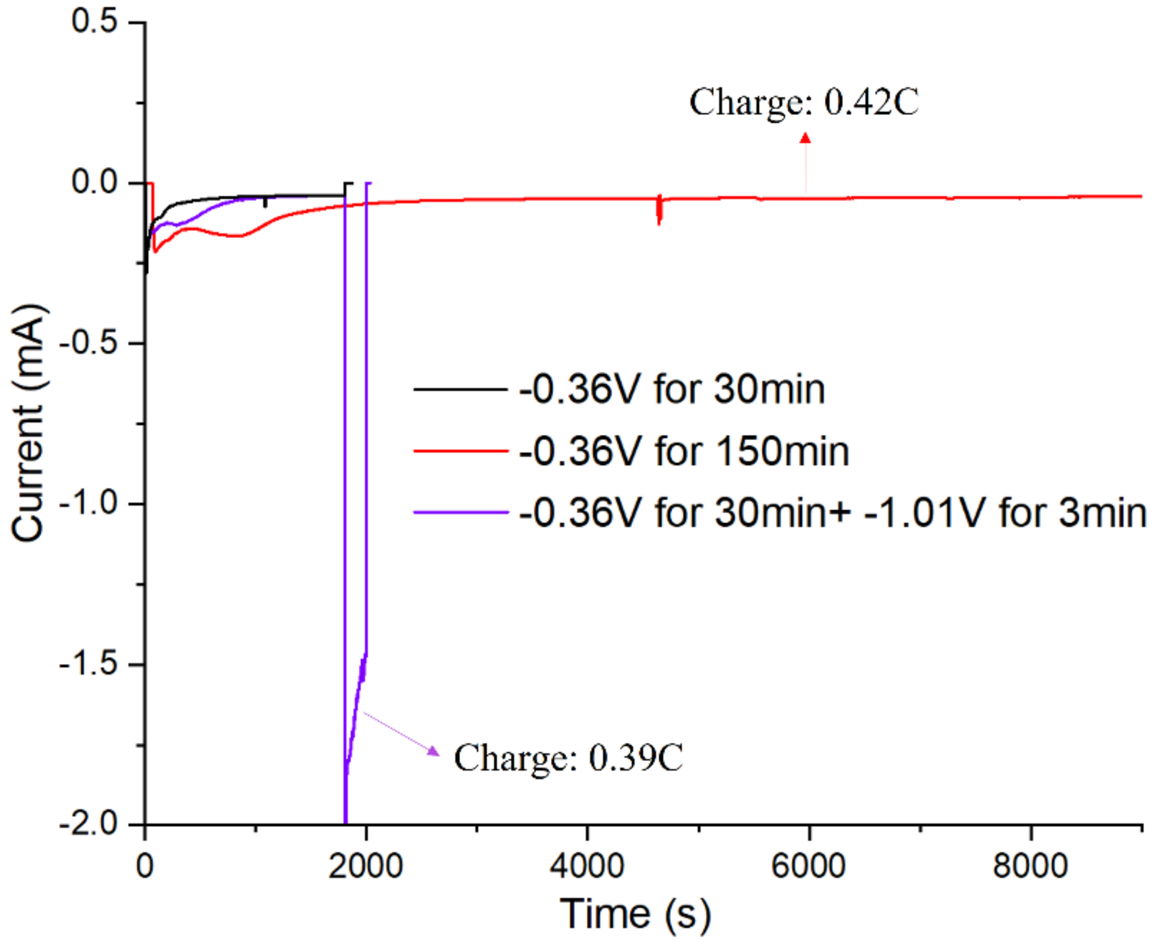

Figure S18 Current density recorded during cathodization before CV experiments in **Figure 3**a.

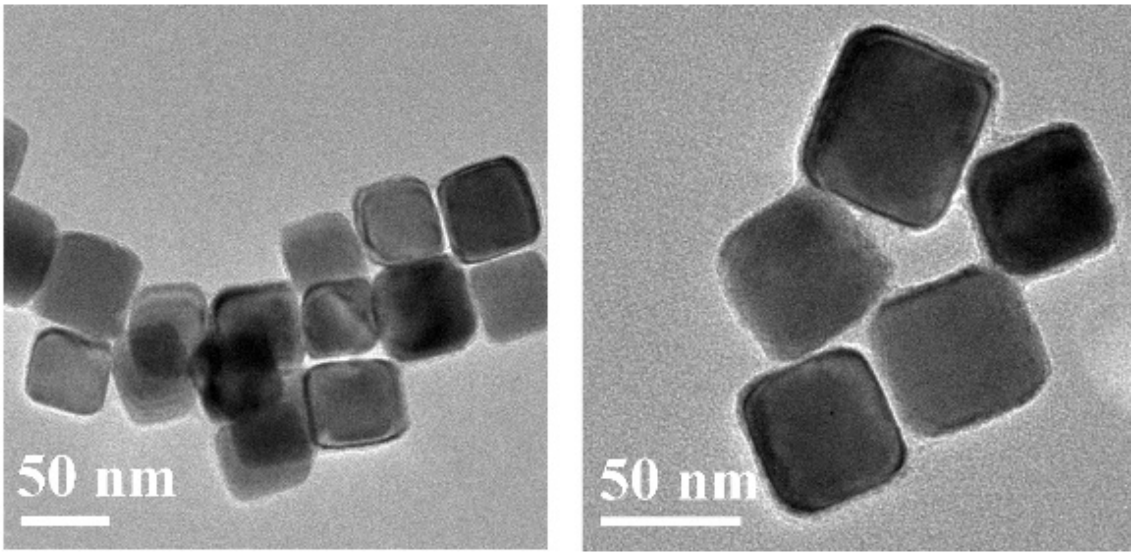

Figure S19 Morphology of the CuNCs after 150min reaction at -0.36V_{RHE} in Ar saturated 0.1M $KHCO_3$ imaged with Ex-situ TEM **(Table S1,** entry 25).

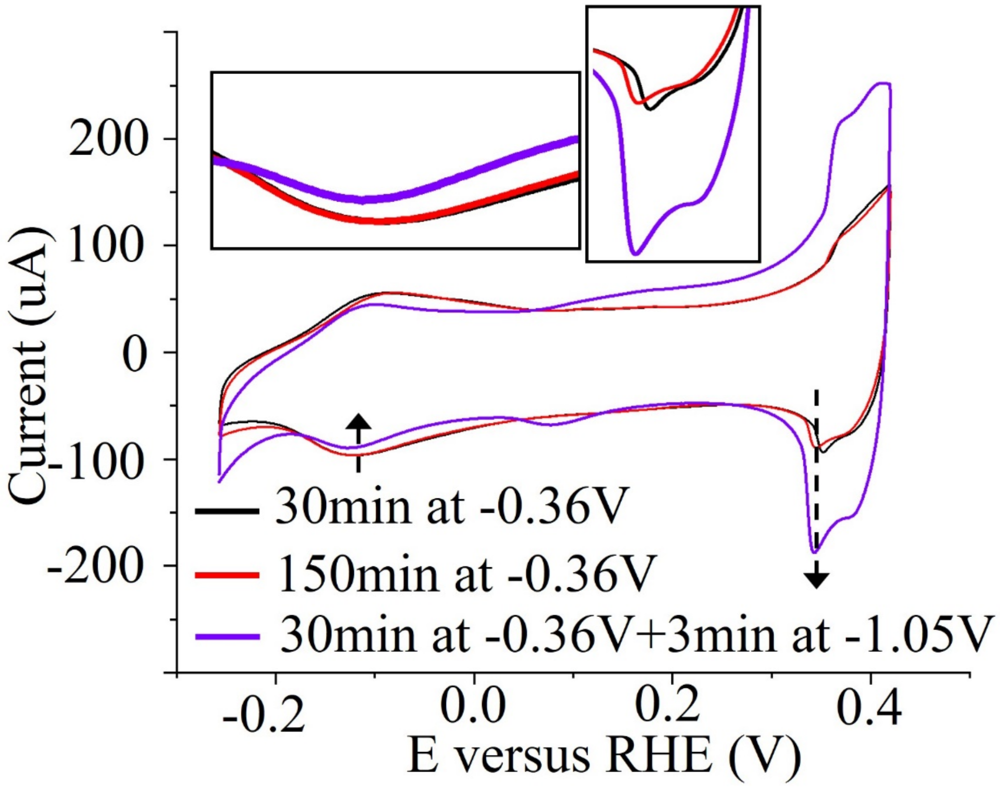

Figure S20 Repeated Voltammograms of Cu nanocubes after 30 mins (black curve) and 150 mins (red curve) of reactions at around -0.36V_{RHE} in 0.1M $KHCO_3$, in contrast of the profile after an initial 30-min reaction at around -0.36V_{RHE}, followed by a 3-min reaction at approximately -1.01 V_{RHE}.

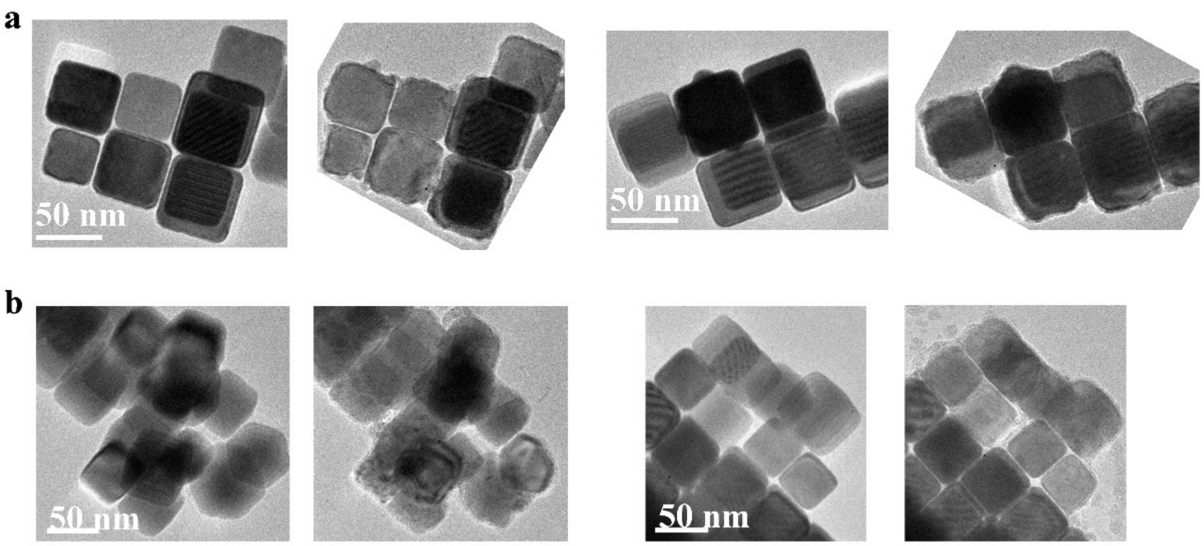

Figure S21 Morphology evolution of Cu cubes imaged with IL-TEM **(a)** before and after 30min reaction at -0.36V_{RHE} **(Table S1,** entry 20).and **(b)** 30min reaction at -0.36V_{RHE} followed by 3min at -1.01V in Ar saturated 0.1M KHCO3 **(Table S1,** entry 26).

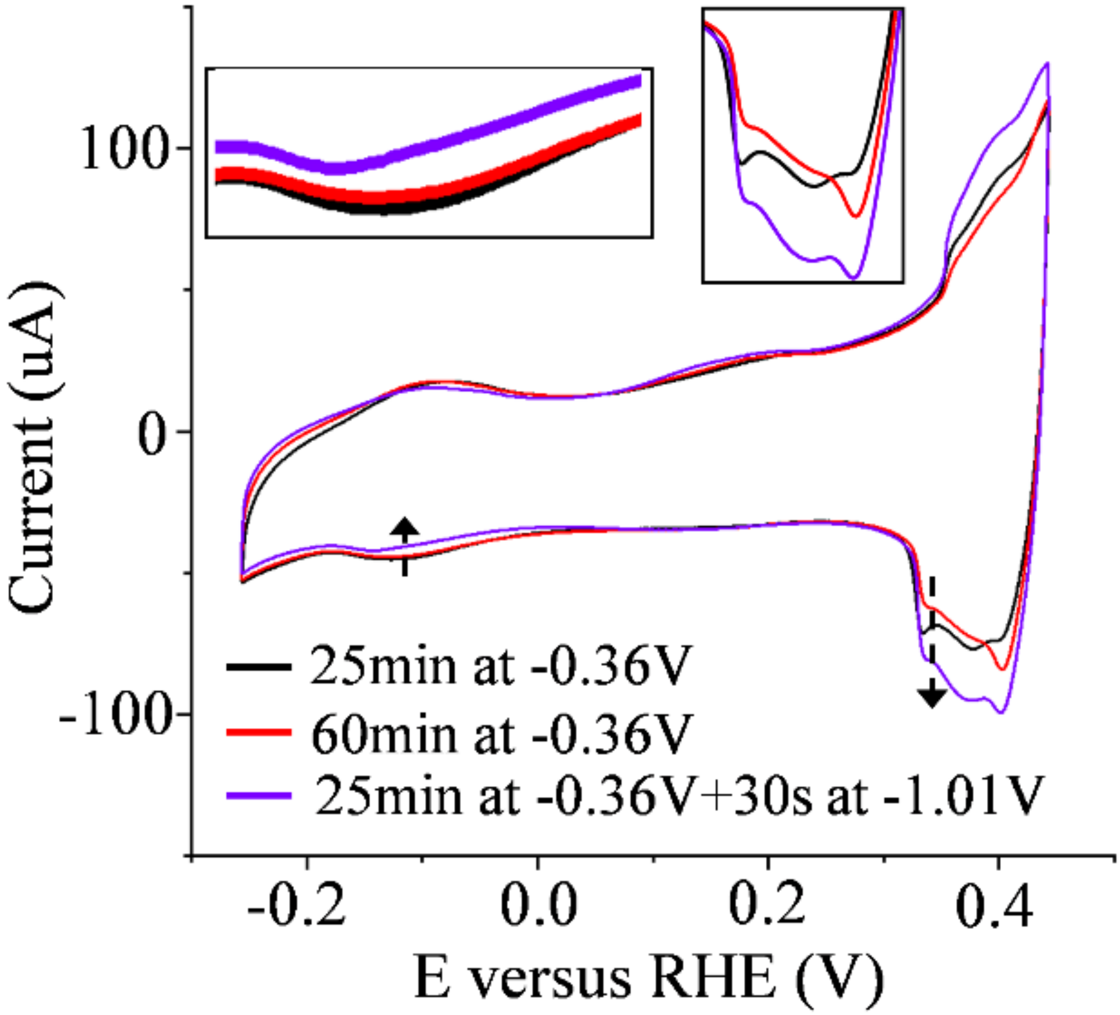

Figure S22 The voltammetric profiles of Cu nanocubes after 25 mins (black curve) and 60 mins (red curve) of reactions at around -0.36V_{RHE} in 1M Ar-saturated KOH are presented. The purple curve depicts the profile after an initial 25-min reaction at around -0.36V_{RHE}, followed by a 30-seconds reaction at roughly -1.01 V_{RHE} **(Table S1,** entries 27-28).

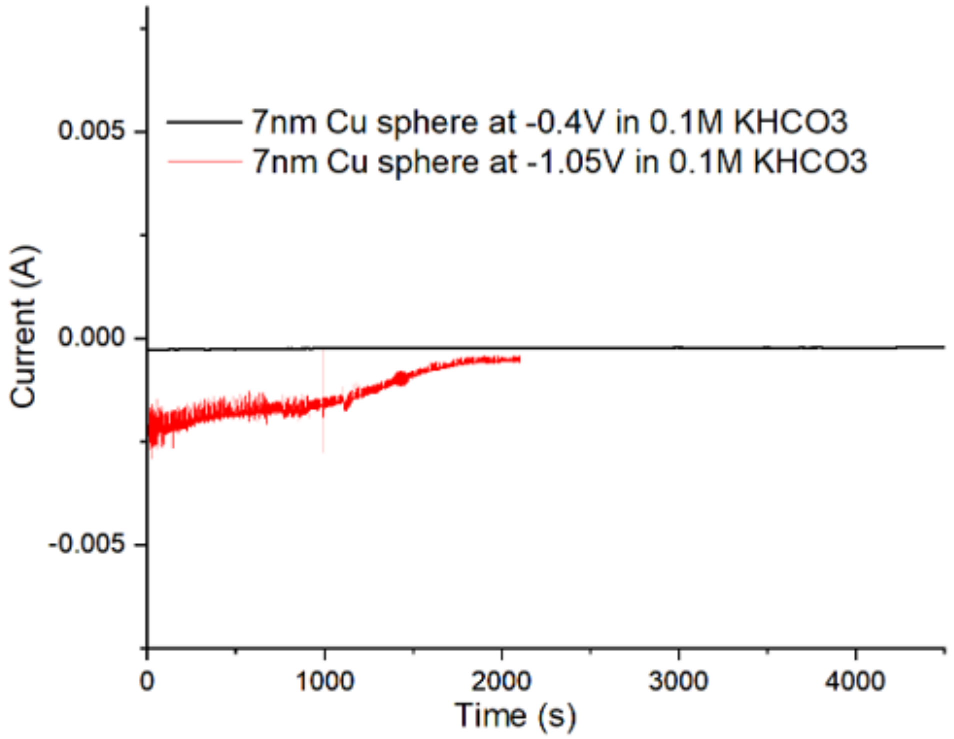

Figure S23 Current of the 7 nm NPs at -1.05 V_{RHE} and -0.4V_{RHE} measured in in situ XAFS cell configuration.

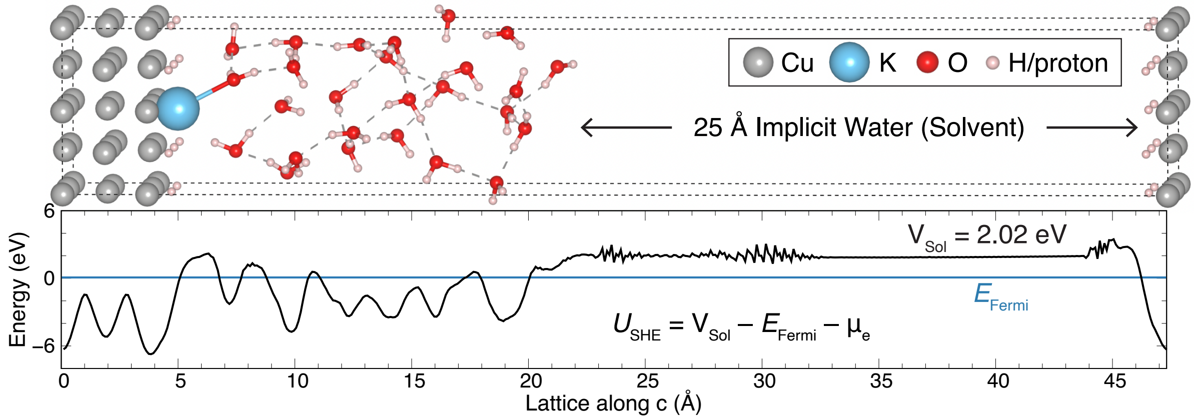

Figure S24 The interface model used in the Ab initio molecular dynamics (AIMD) simulation of Cu dissolution at cathodic potentials, consisting of protonated copper surface, potassium ion, and water molecules (copper in grey, proton/hydrogen in white, potassium in blue, and oxygen in red), a 25 Å implicit bulk water region is added on top of the explicit water molecules along *c* direction (top). Local electrostatic potential of the water solvent in this model is determined from the implicit water region, and the copper electrode potential versus standard hydrogen electrode (SHE) is calculated from its Fermi level (bottom).

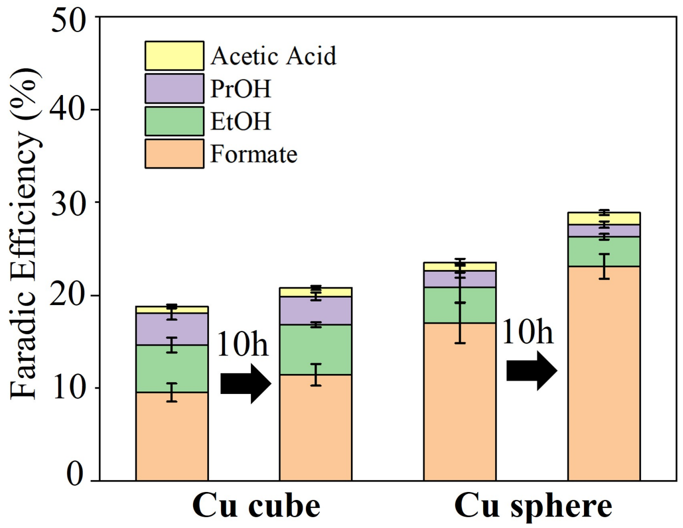

Figure S25 Liquid phase product faraday efficiencies over 10h CO_2RR in 0.1M $KHCO_3$ of the Cu nanocubes at -1.1 V_{RHE} (**Table S1**, entries 1 and 31). The figure show liquid phase products only.

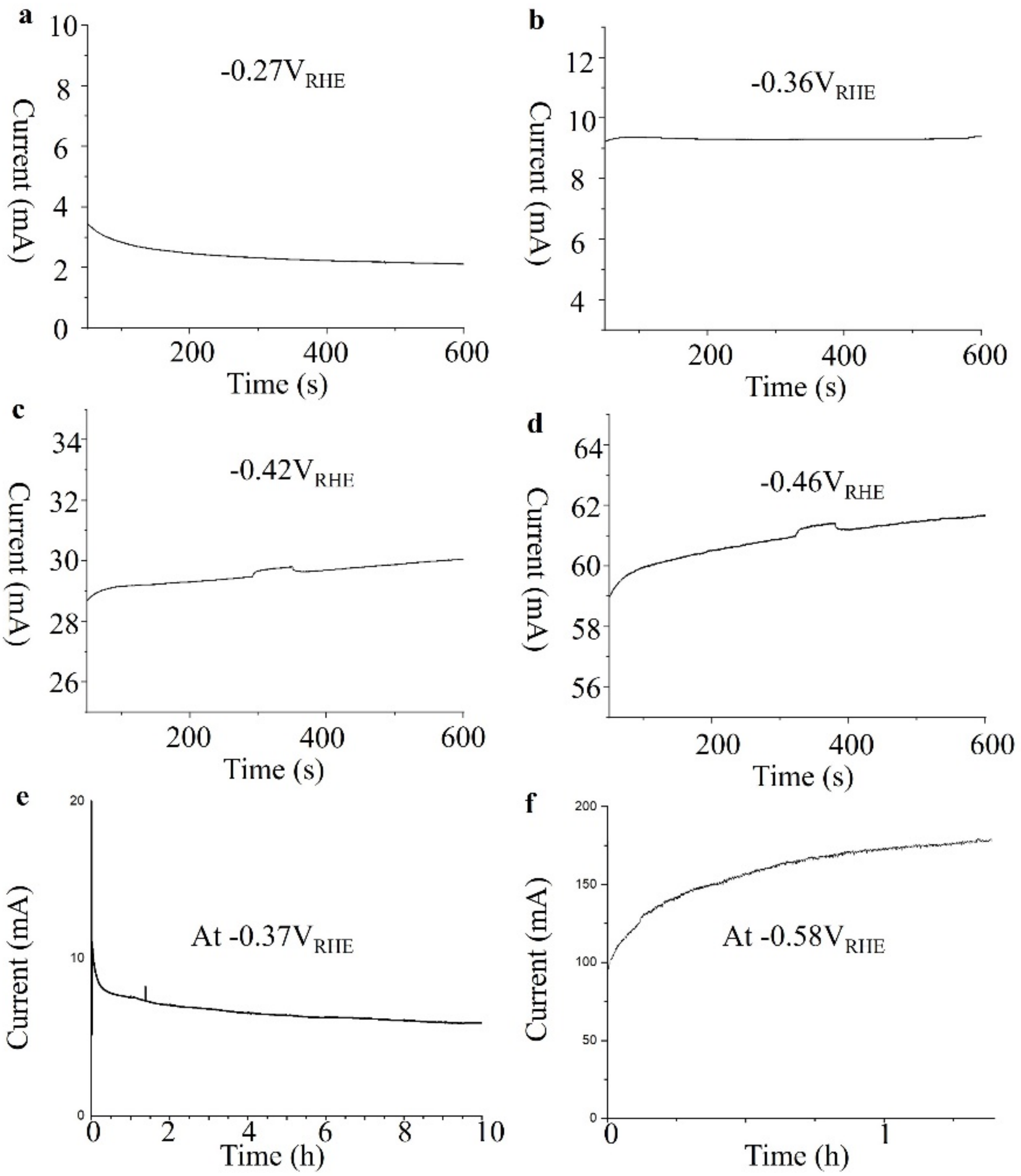

Figure S26 Online current of CORR using Cu nanocubes and 1M KOH at different cathodic potentials. The CORR was carried out using the same electrode loaded with Cu nanocubes, with **(a)** 10 mins at -0.27V$_{RHE}$. and then (**b**) 10 mins at -0.36V$_{RHE}$, followed by **(c)** 10 mins at -0.42V$_{RHE}$ and finally **(d)** 10 mins at -0.46V$_{RHE}$. Current of CORR using two other electrodes of Cu nanocubes at -0.37V over 10h (**e**) and at -0.58V over 80min (**f**), showing consistent trend **(Table S1**, entries 32-37).

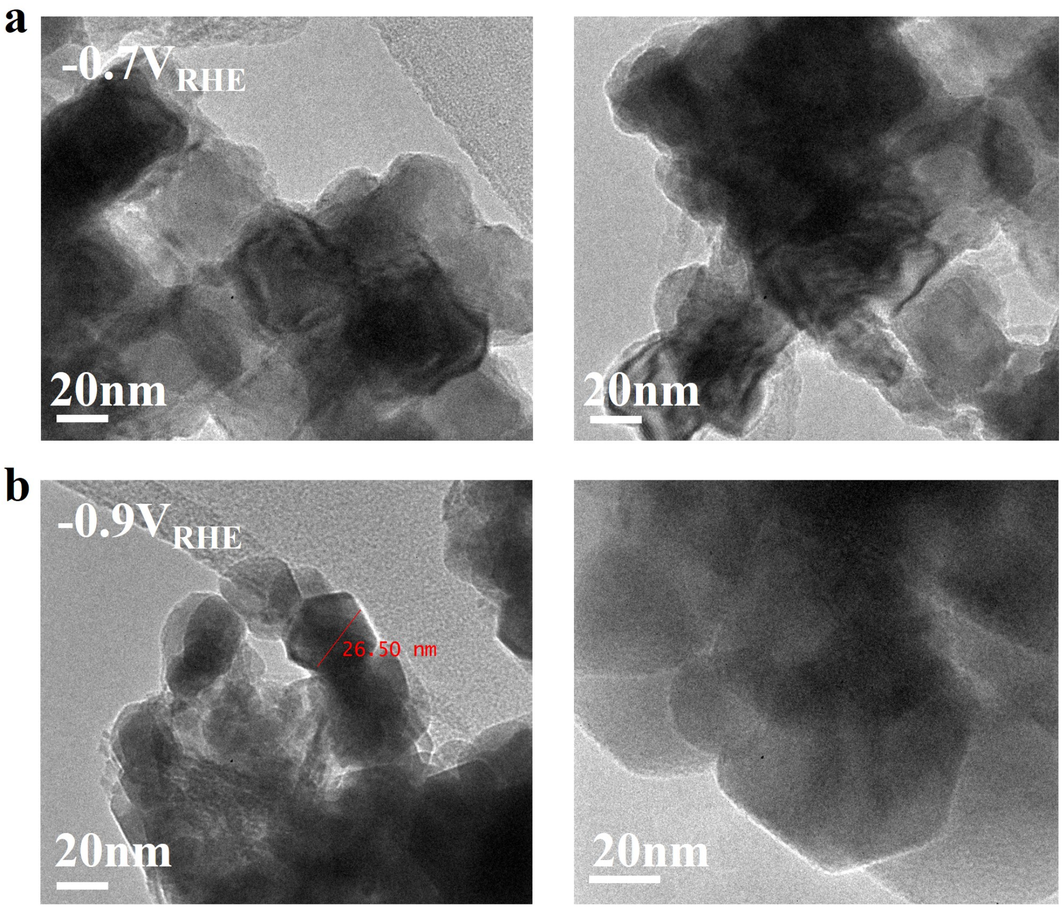

Figure S27 Ex situ TEM bright field images of the CuNCs after 75min CORR (**a**) at -0.7V_{RHE} and 40min CORR (**b**) at -0.9V_{RHE}. The shape of Cu NCs were observed to degrade after the reaction, which is quite different from the stabilized morphology observed on Cu NCs after CORR at -0.37V_{RHE}**, Table S1**, entries 38-39.

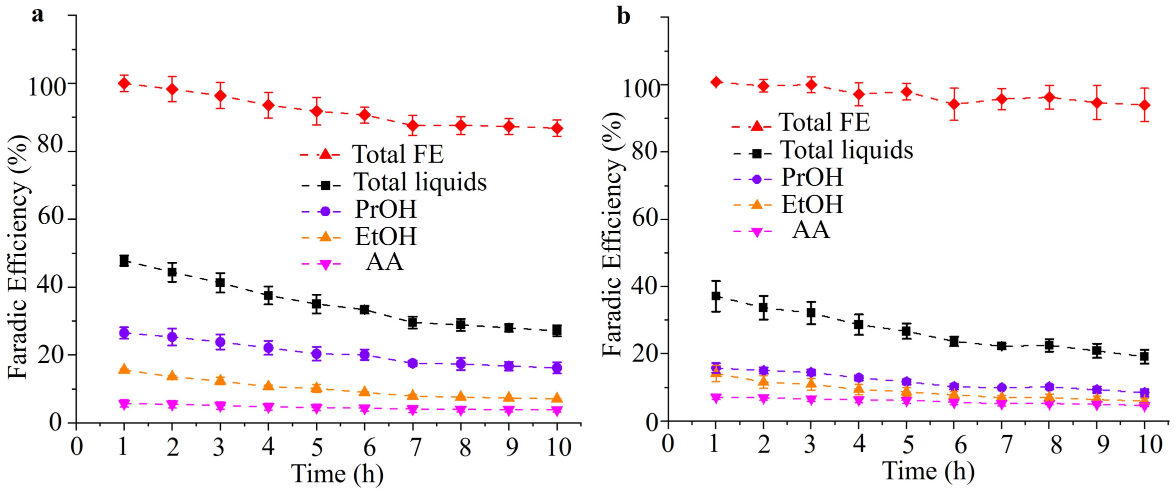

Figure S28 CORR Faraday efficiency as a function of time for (a) Cu nanocubes **(Table S1**, entry 36) and (b) 25 nm Cu spheres **(Table S1**, entry 40).

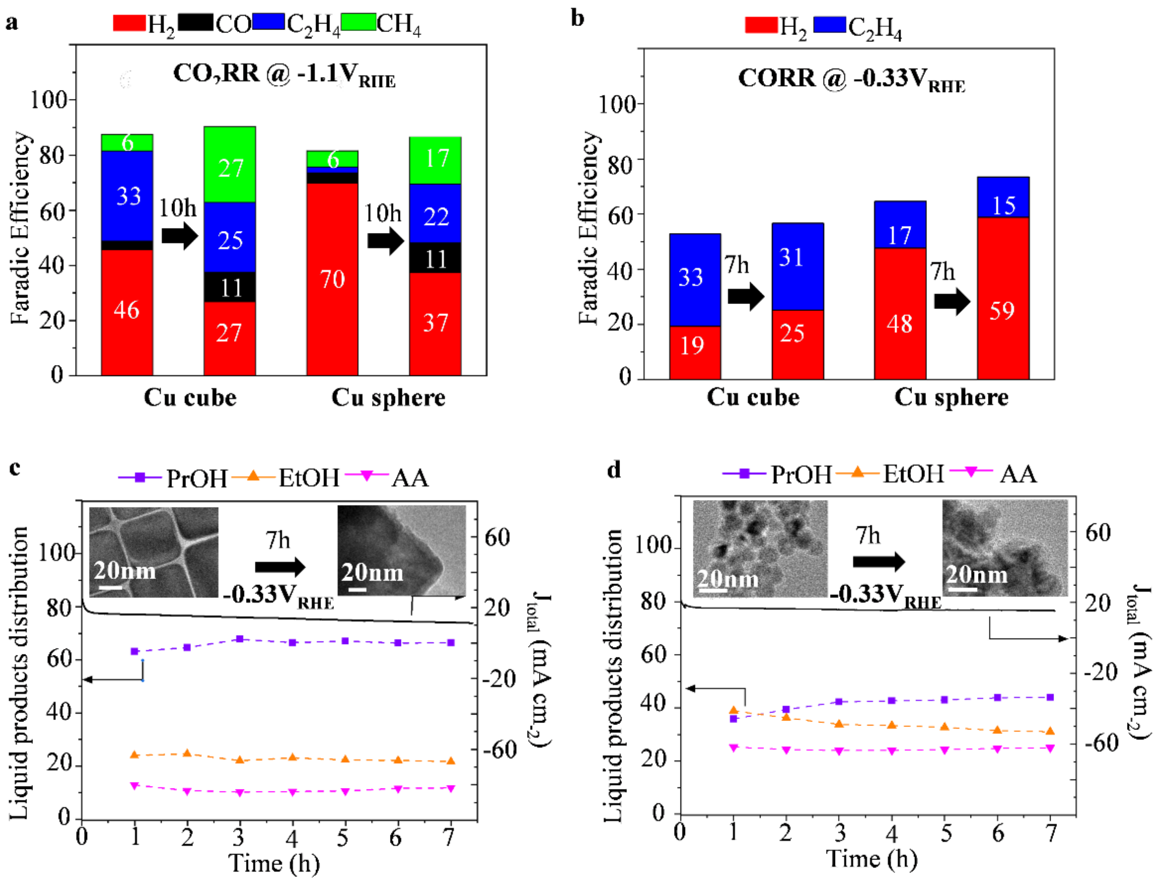

Figure S29 Comparing the long-term morphology-dependent gas products Faradic Efficiency distribution between Cu nanocubes and 7nm spherical Cu nanoparticles in the case of (**a**) CO_2RR at -1.1V_{RHE} and (**b**) CORR test at -0.33V_{RHE}. Time-on-line CORR performance of **(c)** Cu nanocubes **(Table S1**, entry 41) and (**d**) 7nm spherical Cu nanoparticles at -0.33V_{RHE} in 1 M KOH **(Table S1**, entry 42). The time-online current and the liquid products distribution are plotted. Insets show *ex situ* TEM bright field images of the corresponding sample in each case before and after the CORR.

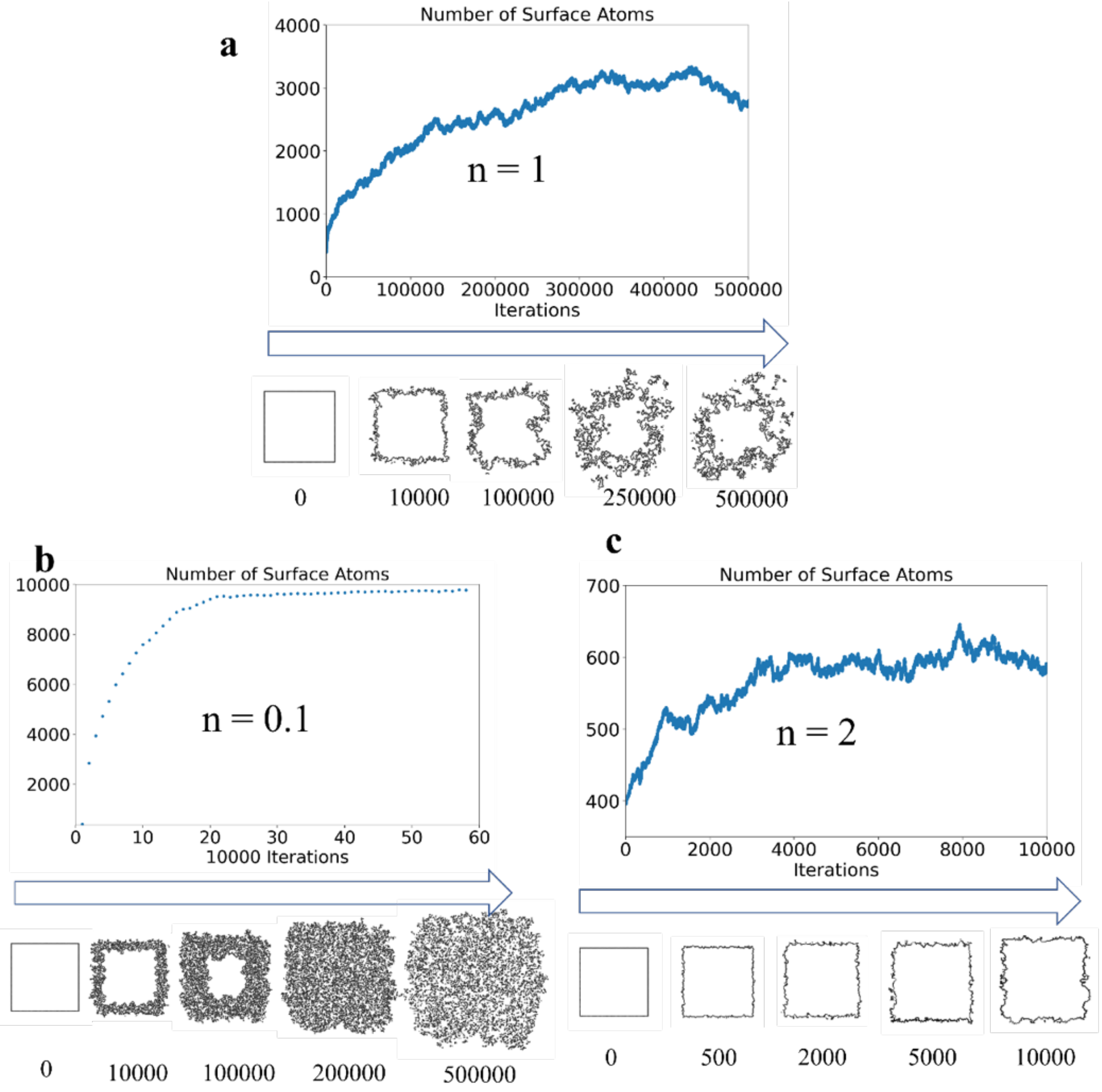

Figure S30 Conceptual simulations of cathodic corrosion process using a 2D lattice of 100 by 100 atoms, demonstrating that an equilibrium could be achieved. Every iteration an atom from the surface will be randomly removed. And then a ball will be randomly added back, simulating the etching and redepositing of Cu atoms. The probabilities for an atom to be removed depend on its coordination number C_i. The lower the coordination number, more likely it will be removed. The probability P_i^a for an atom i to be removed at each iteration is:

$$P_i^a = (4 - C_i)^n \Big/ \sum_i (4 - C_i)^n$$

The probabilities for a surface vacancy to be filled also depend on the coordination number C_i, higher the coordination number, more likely it will be filled. The probability P_i^v for an atom i to be removed at each iteration is:

$$P_i^v = (C_i)^n \Big/ \sum_i (C_i)^n$$

A parameter n is defined as the power to control how important is the coordination number in the random process. We define the max coordination number as 4 in this 2D lattice. The plots of a number of surface atoms (defined by atoms with $C_i < 4$) as a function of the iteration number, and the corresponding snapshots of the 2D cube at different iteration stages, with different power parameters are shown. Panels **a**, **b** and **c** in **Figure S30** show the cases for **n = 1, 0.1** and **2**, respectively.

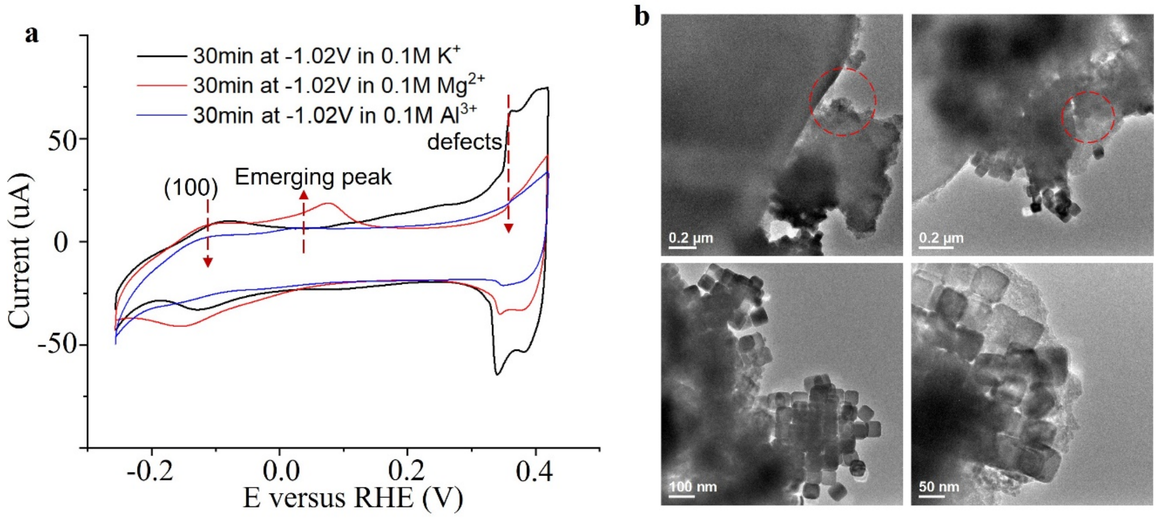

Fig S31 (**a**) Voltametric profiles of the Cu nanocubes treated in Ar saturated 0.05M K_2SO_4 (black), 0.1M $MgSO_4$ (red) and 0.05M $Al_2(SO4)_3$ (purple) at -1.02V_{RHE} for 30min (**Table S1**, entries 43-45). With a higher valence of the cations, Cu related signal in CV was more suppressed. (**b**) TEM images of the Cu nanocubes treated in 0.05M $Al_2(SO4)_3$ electrolyte at -1.02V_{RHE} for 30min. The cubic shape was well preserved due to the protection of Cu surface by $Al(OH)_3$ precipitates or due to the absence of cathodic corrosion in Al^{3+} electrolyte. The regions marked by red circles were possible precipitated Al species covering the Cu nanocubes.

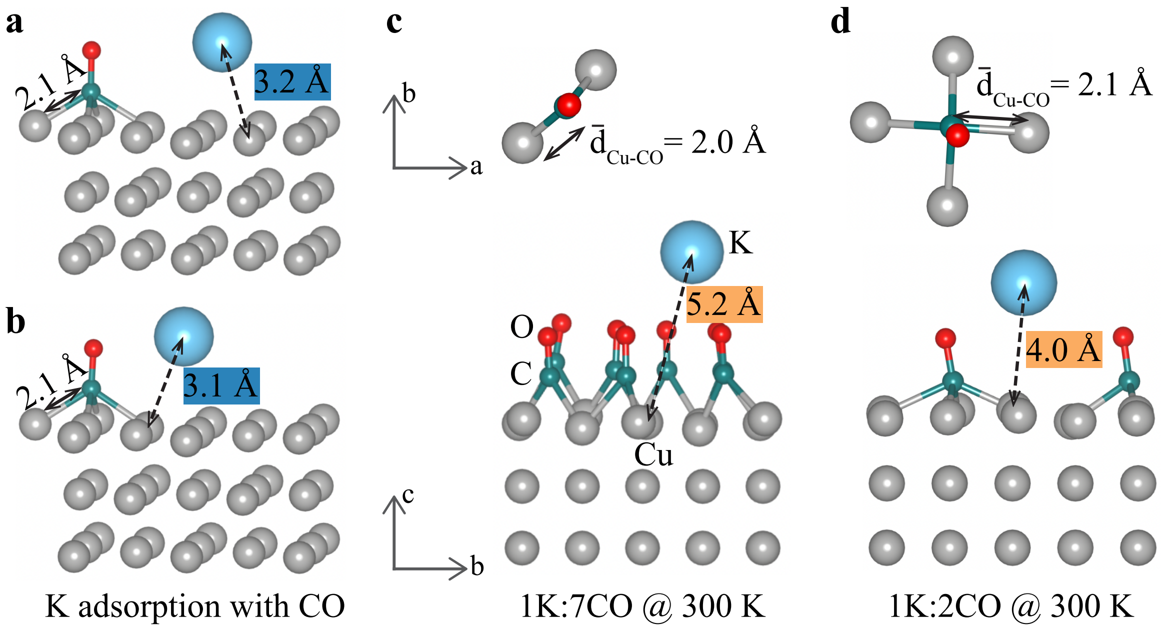

Figure S32. K and CO configurations at the Cu electrode under cathodic conditions from first-principles structure optimization and molecular dynamics (AIMD). (a, b) Optimized adsorption structures for K with the presence of CO, one with K far away from CO (a), one with K close to CO (b). (c) One K and seven CO at the Cu-water interface, temperature ramped to 300 K in AIMD. (d) One K and two CO at the Cu-water interface, temperature ramped to 300 K in AIMD. The K-Cu distance indicating adsorption and (partial) desorption are marked in blue and orange, respectively. For clarity, all explicit water molecules are concealed in (a-d).

Detailed description of the simulation

Additional ab-initio molecular dynamics (AIMD) and structural optimization calculations for the process of extracting Cu at the Cu-water interface with both K^+ and CO present in the reaction. Cathodic conditions are implemented by adding extra electrons into the systems. In the AIMD simulations, temperature was ramped to 300 K at a speed of 0.1 K per femtosecond.

Figure S32 shows the optimized structures for K adsorption and structures from AIMD at 300 K. The K-Cu distance or bond length are marked with dashed double headed arrows, and shaded in blue for K adsorption and in orange for desorption. The Cu-C bond lengths are marked with solid double headed arrows.

In **Figures S32**(a-b), the optimized geometries show K-Cu bond lengths of 3.1-3.2 Å. The initial structures for (a) and (b) were arranged such that the K ion is at the surface sites farthest from and closest to the CO site, respectively. After optimization, the two distinct adsorption configurations show almost the same K-Cu distance. This result provides the standard K-Cu bond length for K adsorption in the presence of CO. The optimized structures also show that CO is adsorbed at a hollow (four-fold) site on the Cu surface, with an average Cu-C bond length of 2.1 Å.

Figure S32(c-d) shows the equilibrated geometry at a lower K:CO ratio of 1:7 (c) and a higher ratio of 1:2 (d), calculated from AIMD at 300 K. In both cases, K and CO were initially put at adsorption sites on the Cu surface before the AIMD calculations. In the equilibrated structures, the K-Cu distances show substantial increase compared to the case of K adsorption (Figure R(a-b)). This indicates that the K ion desorbs from the Cu surface and the K-Cu interaction

becomes much weaker than adsorption. In the meantime, the CO molecules remain adsorbed, as indicated by the bond length of below 2.1 Å.

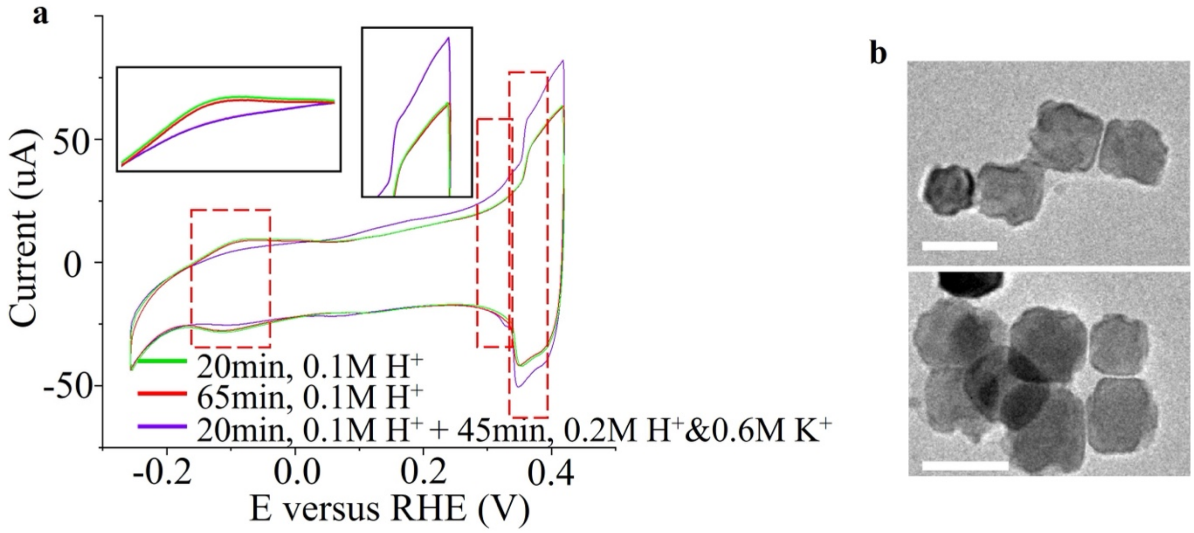

Figure S33 (a) Voltammetric profiles of the Cu nanocubes recorded after 20 mins (green curve) and 65 mins (red curve) of reaction at about -1.05V_{RHE} in Ar saturated 0.05M H_2SO_4 (**Table S1**, entry 7). The purple curve represents the case where the Cu nanocubes first undergo 20 mins of treatment in Ar saturated 0.05M H_2SO_4, followed by subsequent 45 mins of reaction at about -1.05V_{RHE} (purple curve) in Ar saturated 0.1M H_2SO_4 +0.3M K_2SO_4 (**Table S1**, entry 46) (b) TEM images of Cu NCs after 1h treatment in 0.1M H2SO4 +0.3M K_2SO_4. The scale bar represents 50nm. After additional 45min cathodic treatment in K+ containing electrolyte, the defects related signal increases and 100 facet related signal decreases. The scale bars represent 50 nm.

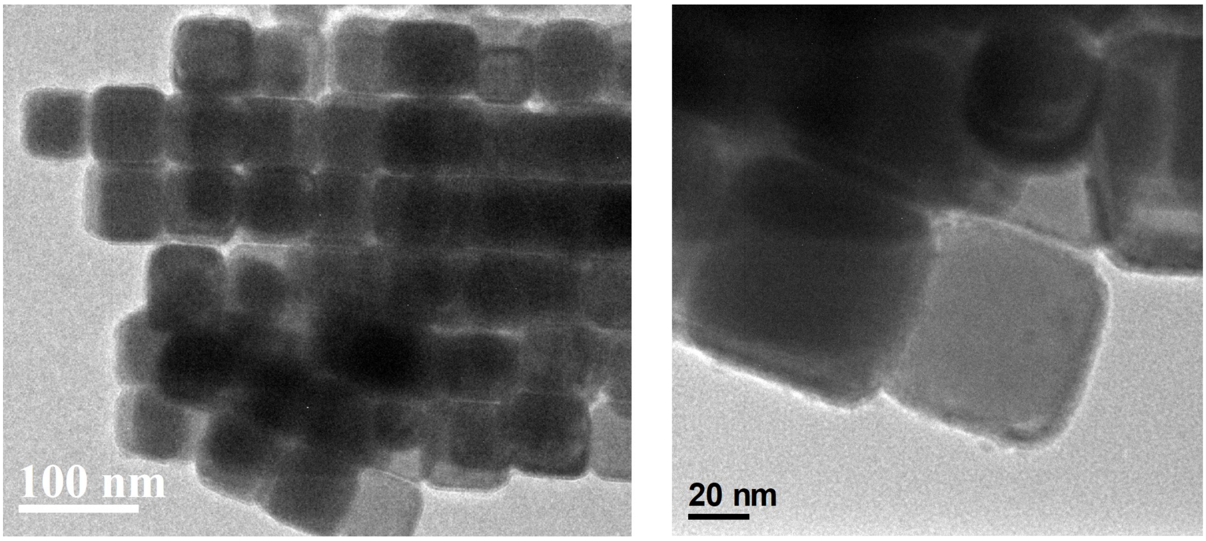

Figure S34 Representative TEM bright field images of Cu nanocubes after Ar plasma at 30W for 60s.

Table S1 Summary of the experimental conditions investigated in this study, detailing the methods employed and the observation of alkali cation-induced cathodic corrosion. Instances where significant morphological changes of Cu were not observed are highlighted with 'No' in red in the corresponding columns representing the observation techniques. 'n.a.' indicates that data is not available.

#	Electrochemical Treatment Conditions					IL-TEM	CV†	Ex situ TEM
	Potential	Reactor	Gas	Electrolyte	Note	Significant morphology changes of Cu?		
1	$\sim -1.1\ V_{RHE}$	H-Cell	CO_2	0.1M $KHCO_3$ pH=6.8	Initial long-term CO_2RR stability test (10h) with Cu nanocubes. Figures S5, S23, 6	n.a.	n.a.	Yes
2	$\sim -1.1\ V_{RHE}$	Single Cell	CO_2	0.1M $KHCO_3$	IL-TEM to initially identify morphological changes beyond the reduction of surface oxides Figures 1, S6, S7	Yes	n.a.	Yes
3	$\sim -1.1\ V_{RHE}$	Single Cell	Ar	0.1M $KHCO_3$ pH=8.6	Having CO_2 is not a necessary factor for the observed morphological change Figures 2, S7, S9, S10, S11, S13	Yes	Yes	Yes
4	$\sim 0\ V_{RHE}$	Single Cell	Ar	0.1M $KHCO_3$	Additional experiment to show that we did not just observe the reduction of surface oxides Figures S7, S8	No	n.a.	n.a.
5	$\sim -1.1\ V_{RHE}$	Single Cell	Ar	0.1M K_2SO_4 pH=7	The effect of different anions. Figures S11	Yes	n.a.	Yes
6	$\sim -1.1\ V_{RHE}$	Single Cell	Ar	0.1M K_2CO_3 pH=11.6		Yes	n.a.	Yes
7	$\sim -1.1\ V_{RHE}$	Single Cell	Ar	0.05M H_2SO_4 pH=1.16	The effect of K+ cation Figures 2, S12	No	No	n.a.
8	$\sim -1.1\ V_{RHE}$	Single Cell	Ar	0.05M H_2SO_4 + 0.3M K_2SO_4 pH=1.28		Yes	Yes	n.a.
9	$\sim -1.1\ V_{RHE}$	Single Cell	Air	0.05M H_2SO_4	To purposely reoxidize Cu and confirm that the phenomenon we observed is very different than oxidation/reduction of Cu Figure S12	Yes	Yes	n.a.
10	$\sim -1.1\ V_{RHE}$	Single Cell	Ar	0.5M $KHCO_3$	Changing K+ concentration Figure S13	Yes	Yes	n.a.
11	$\sim -1.1\ V_{RHE}$	Single Cell	Ar	1.5M $KHCO_3$		Yes	Yes	n.a.
12	$\sim -1.1\ V_{RHE}$	Single Cell	Ar	0.2M Li_2SO_4	Constant potential experiments using different alkali cations Figure S14	n.a.	The reference for the group	n.a.
13	$\sim -1.1\ V_{RHE}$	Single Cell	Ar	0.2M Na_2SO_4		n.a.	Yes	n.a.
14	$\sim -1.1\ V_{RHE}$	Single Cell	Ar	0.2M K_2SO_4		n.a.	Yes	n.a.
15	$\sim -1.1\ V_{RHE}$	Single Cell	Ar	0.2M Cs_2SO_4		n.a.	Yes	n.a.

16	~ -1.27 V_{RHE}	Single Cell	Ar	0.2M Li_2SO_4	Constant current experiments using different alkali cations Figure S15	n.a.	Yes	n.a.
17	~ -1.1 V_{RHE}	Single Cell	Ar	0.2M Cs_2SO_4		n.a.	Yes	n.a.
18	~ -0.92 V_{RHE}	Single Cell	Ar	0.05M H_2SO_4		n.a.	The reference for the group	n.a.
19	~ -1.5 V_{RHE}	Single Cell	Ar	0.05M H_2SO_4+0.3M K_2SO_4		n.a.	Yes	n.a.
20	~-0.36 V_{RHE}	Single Cell	Ar	0.1M $KHCO_3$	Looking for the threshold potential Figures 3, S9, S10, S16, S19	No	No	n.a.
21	~ -0.4 V_{RHE}	Single Cell	Ar	0.1M $KHCO_3$		n.a.	No	n.a.
22	~ -0.6 V_{RHE}	Single Cell	Ar	0.1M $KHCO_3$		n.a.	Yes	n.a.
23	~-0.8 V_{RHE}	Single Cell	Ar	0.1M $KHCO_3$		n.a.	Yes	n.a.
24	~ -1.3 V_{RHE}	Single Cell	Ar	0.1M $KHCO_3$		n.a.	Yes	n.a.
25	~ -0.36 V_{RHE} (30mins + 120mins)	Single Cell	Ar	0.1M $KHCO_3$	Additional experiments to confirm the effect of the electrodepotential<br><br>Figures S9, S10, 3 , S17, S18, S19, S20	No	No	No
26	~ -0.36 V_{RHE} (30 mins) + ~ -1 V_{RHE} (3min)	Single Cell	Ar	0.1M $KHCO_3$		Yes	Yes	n.a.
27	~ -0.36 V_{RHE} (25mins + 35 mins)	Single Cell	Ar	1M KOH		n.a.	No	n.a.
28	~ -0.36 V_{RHE} (25mins) + ~ ~1 V_{RHE} (30 secs)	Single Cell	Ar	1M KOH pH=14		n.a.	Yes	n.a.
29	~ -0.4 V_{RHE}	In situ cell	CO_2	0.1M $KHCO_3$	In situ XAFS experiment Spherical 7nm Cu nanoparticles Figures 4, S21	n.a.	n.a.	n.a.
30	~ -1.1 V_{RHE}	In situ cell	CO_2	0.1M $KHCO_3$		n.a.	n.a.	n.a.
31	~ -1.1 V_{RHE}	H-Cell	CO_2	0.1M $KHCO_3$	10 hour CO_2RR with Spherical 25nm Cu nanoparticles Figures 6, S23	n.a.	n.a.	Yes
32	~ -0.27V_{RHE}	GDE, flow cell	CO	1M KOH	Comparison of variation trend in CORR current using Cu nanocubes between different cathodic potentials<br><br>Figures S24	n.a.	n.a.	n.a.
33	~ -0.37 V_{RHE}	GDE, flow cell	CO	1M KOH		n.a.	n.a.	n.a.
34	~ -0.42 V_{RHE}	GDE, flow cell	CO	1M KOH		n.a.	n.a.	n.a.
35	~ -0.46 V_{RHE}	GDE, flow cell	CO	1M KOH		n.a.	n.a.	n.a.
36	~ -0.37 V_{RHE}	GDE, flow cell	CO	1M KOH	Long-term stability test (10h) with 40nm Cu nanocubes Figures 6, S24, S26	n.a.	n.a.	No
37	~ -0.58 V_{RHE}	GDE, flow cell	CO	1M KOH	Long-term stability test (80 mins) with 40nm Cu nanocubes Figures S24	n.a.	n.a.	n.a.
38	~ -0.7 V_{RHE}	GDE, flow cell	CO	1M KOH	Post-reaction observation after CORR at more negative potentials<br><br>Figures S25	n.a.	n.a.	Yes
39	~ -0.9 V_{RHE}	GDE, flow cell	CO	1M KOH		n.a.	n.a.	Yes

40	~ -0.37 V_{RHE}	GDE, flow cell	CO	1M KOH	Long-term CORR stability test (10h) with 25 nm Cu spherical nanoparticles Figures 6, S26	n.a.	n.a.	No
41	~ -0.33 V_{RHE}	GDE, flow cell	CO	1M KOH	Additional long-term stability test (7h) with 40nm Cu nanocubes Figures S27	n.a.	n.a.	No
42	~ -0.33 V_{RHE}	GDE, flow cell	CO	1M KOH	Additional long-term stability test (7h) with 7nm nanoparticles Figures S27	n.a.	n.a.	Yes (possibly due to agglomerat ion)
43	~ -1 V_{RHE}	Single Cell	Ar	0.05M K_2SO_4	To check the effect of group II and III cations, at pH level is 3<br><br>Figure S29	n.a.	Possible precipitation of hydroxides exclude meaningful conclusions, possibly due to local basic conditions, despite the overall pH of the electrolyte is about 3.	
44	~ -1 V_{RHE}	Single Cell	Ar	0.1M $MgSO_4$		n.a.		
45	~ -1 V_{RHE}	Single Cell	Ar	0.05M $Al_2(SO_4)_3$		n.a.		
46	~ -1.1 V_{RHE}	Single Cell	Ar	0.1M H_2SO_4 + 0.3M K_2SO_4 pH=0.98	Additional experiment to confirm the pH of the electrolyte does not play major role in whether or not the alkali-cation induced cathodic corrosion will be observed in Cu	n.a.	Yes	Yes

†For CV experiment, unless specified otherwise, the results were compared to a baseline established by pre-treating the catalysts at the same voltages for 20-30 mins to make sure the surface oxides were removed.

Table S2 Selective entries from Table S1 showcasing the pH conditions of electrolytes (from more basic to more acidic conditions) tested for Cu NCs around -1.1V_{RHE}, along with the indication of whether alkali cation-induced cathodic corrosion occurred or not. Entries correspond to their respective entry numbers in **Table S1**.

#	Electrochemical Treatment Conditions					IL-TEM	CV†	Ex situ TEM
	Potential	Reactor	Gas	Electrolyte	Note	Significant morphology changes of Cu?		
28	~ -0.36 V_{RHE} (25mins) + ~ ~1 V_{RHE} (30 secs)	Single Cell	Ar	1M KOH pH=14	Additional experiments to confirm the effect of the electrodepotential<br><br>Figures S9, S10, 3 , S17, S18, S19, S20	n.a.	Yes	n.a.
6	~ -1.1 V_{RHE}	Single Cell	Ar	0.1M K_2CO_3 pH=11.6	The effect of different anions. Figures S11	Yes	n.a.	Yes
3	~ -1.1 V_{RHE}	Single Cell	Ar	0.1M $KHCO_3$<br><br>pH=8.6	Having CO_2 is not a necessary factor for the observed morphological change Figures 2, S7, S9, S10, S11, S13	Yes	Yes	Yes
5	~ -1.1 V_{RHE}	Single Cell	Ar	0.1M K_2SO_4<br><br>pH=7	The effect of different anions. Figures S11	Yes	n.a.	Yes

1	~ -1.1 V_{RHE}	H-Cell	CO_2	0.1M $KHCO_3$<br><br>pH=6.8	Initial long-term CO_2RR stability test (10h) with Cu nanocubes.<br>Figures S5, S23, 6	n.a.	n.a.	Yes
8	~ -1.1 V_{RHE}	Single Cell	Ar	0.05M H_2SO_4 + 0.3M K_2SO_4<br><br>pH=1.28	The effect of K+ cation<br>Figures 2, S12	Yes	Yes	n.a.
7	~ -1.1 V_{RHE}	Single Cell	Ar	0.05M H_2SO_4<br><br>pH=1.16		No	No	n.a.
46	~ -1.1 V_{RHE}	Single Cell	Ar	0.1M H_2SO_4 + 0.3M K_2SO_4<br>pH=0.98	Additional experiment to confirm the pH of the electrolyte does not play major role in whether or not the alkali-cation induced cathodic corrosion will be observed in Cu<br><br>Figure S32	n.a.	Yes	Yes
